# Characterization of joining sites of a viral histone H4 on host insect chromosomes

**DOI:** 10.1371/journal.pone.0177066

**Published:** 2017-05-09

**Authors:** Sunil Kumar, Jin-Kyo Jung, Yonggyun Kim

**Affiliations:** 1 Department of Plant Medicals, Andong National University, Andong, Korea; 2 Division of Crop Cultivation and Environment Research, Department of Central Area Crop Science, National Institute of Crop Science, Rural Development Administration, Suwon, Korea; Institute of Plant Physiology and Ecology Shanghai Institutes for Biological Sciences, CHINA

## Abstract

A viral histone H4 (CpBV-H4) is encoded in a polydnavirus, Cotesia plutellae bracovirus (CpBV). It plays a crucial role in parasitism of an endoparasitoid wasp, *C*. *plutellae*, against diamondback moth, *Plutella xylostella*, by altering host gene expression in an epigenetic mode by its N-terminal tail after joining host nucleosomes. Comparative transcriptomic analysis between parasitized and nonparasitized *P*. *xylostella* by RNA-Seq indicated that 1,858 genes were altered at more than two folds in expression levels at late parasitic stage, including 877 up-regulated genes and 981 down-regulated genes. Among parasitic factors altering host gene expression, CpBV-H4 alone explained 16.3% of these expressional changes. To characterize the joining sites of CpBV-H4 on host chromosomes, ChIP-Seq (chromatin immunoprecipitation followed by deep sequencing) was applied to chromatins extracted from parasitized larvae. It identified specific 538 ChIP targets. Joining sites were rich (60.2%) in AT sequence. Almost 40% of ChIP targets included short nucleotide repeat sequences presumably recognizable by transcriptional factors and chromatin remodeling factors. To further validate these CpBV-H4 targets, CpBV-H4 was transiently expressed in nonparasitized host at late larval stage and subjected to ChIP-Seq. Two kinds of ChIP-Seqs shared 51 core joining sites. Common targets were close (within 1 kb) to genes regulated at expression levels by CpBV-H4. However, other host genes not close to CpBV-H4 joining sites were also regulated by CpBV-H4. These results indicate that CpBV-H4 joins specific chromatin regions of *P*. *xylostella* and controls about one sixth of the total host genes that were regulated by *C*. *plutellae* parasitism in an epigenetic mode.

## Introduction

Polydnaviruses (PDVs) are a group of insect viruses symbiotic to some endoparasitoid wasps belonging to families Braconidae and Ichneumonidae. Depending on host wasp families, PDVs are divided into two genera: *Ichnovirus* (IV) and *Bracovirus* (BV) [1]. PDV genome consists of genome encapsidated into viral particles during replication and nonencapsidated genome [2]. Its nonencapsidated genome appears to be essential to the replication of encapsidated genome. Some gene products of the nonencapsidated genome are likely viral coat proteins [3]. The encapsidated genome in the viral coat is expressed in parasitized host. It plays crucial roles in altering host physiological processes for successful parasitism [4].

An endoparasitoid wasp, *Cotesia plutellae*, specifically parasitizes young larvae of the diamondback moth, *Plutella xylostella*. It has several parasitic factors, including a symbiotic PDV and *C*. *plutellae* bracovirus (CpBV) [5]. Parasitized *P*. *xylostella* larvae exhibit significant physiological alterations, including immunosuppression and delayed larval development [6,7]. CpBV plays a crucial role in parasitism of *C*. *plutellae*. Its genome contains 157 putative open reading frames (ORFs), most of which are expressed in parasitized larvae [8].

CpBV is a bracoviral PDV. Its encapsidated genome shares common genes with other BVs [9]. However, some genes encoded in the genome of CpBV are unique in *Cotesia*-associated BVs [10]. A viral histone H4 is encoded only in *Cotesia*-associated BVs and CpBV-H4 is a viral histone H4 ortholog of CpBV [11]. CpBV-H4 is highly homologous to host histone H4 except an extended N terminal tail which contains 38 amino acids with nine lysine residues [12]. CpBV-H4 suppresses host immunity by inhibiting gene expression of phenoloxidase and antimicrobial peptides [13]. CpBV-H4 expression also inhibits gene expression of insulin-like peptide of host larvae, leading to developmental retardation and hemolymph hypertrehalosemic conditions which are beneficial for symbiotic parasitoid development [14]. However, these physiological alterations by CpBV-H4 are completely lost when it loses its N-terminal extended tail [15]. These findings suggest that CpBV-H4 can modulate host gene expression in an epigenetic mode.

CpBV-H4 joins eukaryotic nucleosomes by interacting with other histone monomers to form an octamer [16]. Using a nonhost, *Tribolium castaneum*, CpBV-H4 has been transiently expressed and subsequently subjected to chromatin immunoprecipitation (ChIP), in which ChIP targets are located on the upstream or downstream of coding DNA sequences (CDSs) annotated as inducible genes [17]. Furthermore, subtractive suppressive hybridization (SSH) analysis performed in natural host, *P*. *xylostella*, has shown that CpBV-H4 suppresses the expression of at least 115 genes, including chromatin remodeling factors that can subsequently alter other gene expression [14]. These previous results suggest that CpBV-H4 may have structural or DNA sequence specificity to joining sites. However, the molecular characters of the incorporation sites of CpBV-H4 on natural host genome of *P*. *xylostella* remain unknown. In addition to SSH analysis, CpBV-H4 might up-regulate or down-regulate host gene expression. Thus, in addition to the previous ChIP assay on non-natural host and SSH on natural host, genome-wide transcriptomic analyses are needed to understand host-parasite molecular interaction that control host gene expression by CpBV-H4. Furthermore, we do not know how much host gene regulation induced by *C*. *plutellae* parasitism could be explained by CpBV-H4.

Therefore, the objective of this study was to determine the incorporation sites of CpBV-H4 on host chromosomes of *P*. *xylostella* parasitized by *C*. *plutellae* by ChIP-Seq analysis. CpBV-H4 was transiently expressed in nonparasitized host followed by ChIP-Seq to confirm the incorporation sites determined in parasitized host. In addition, host genes changed in expression level by CpBV-H4 were determined by comparing the transcriptome of nonparasitized host transiently expressing CpBV-H4 to that of parasitized host using RNA-Seq.

## Results

### Influence of CpBV-H4 on host gene regulation during parasitism

**Fig 1:**
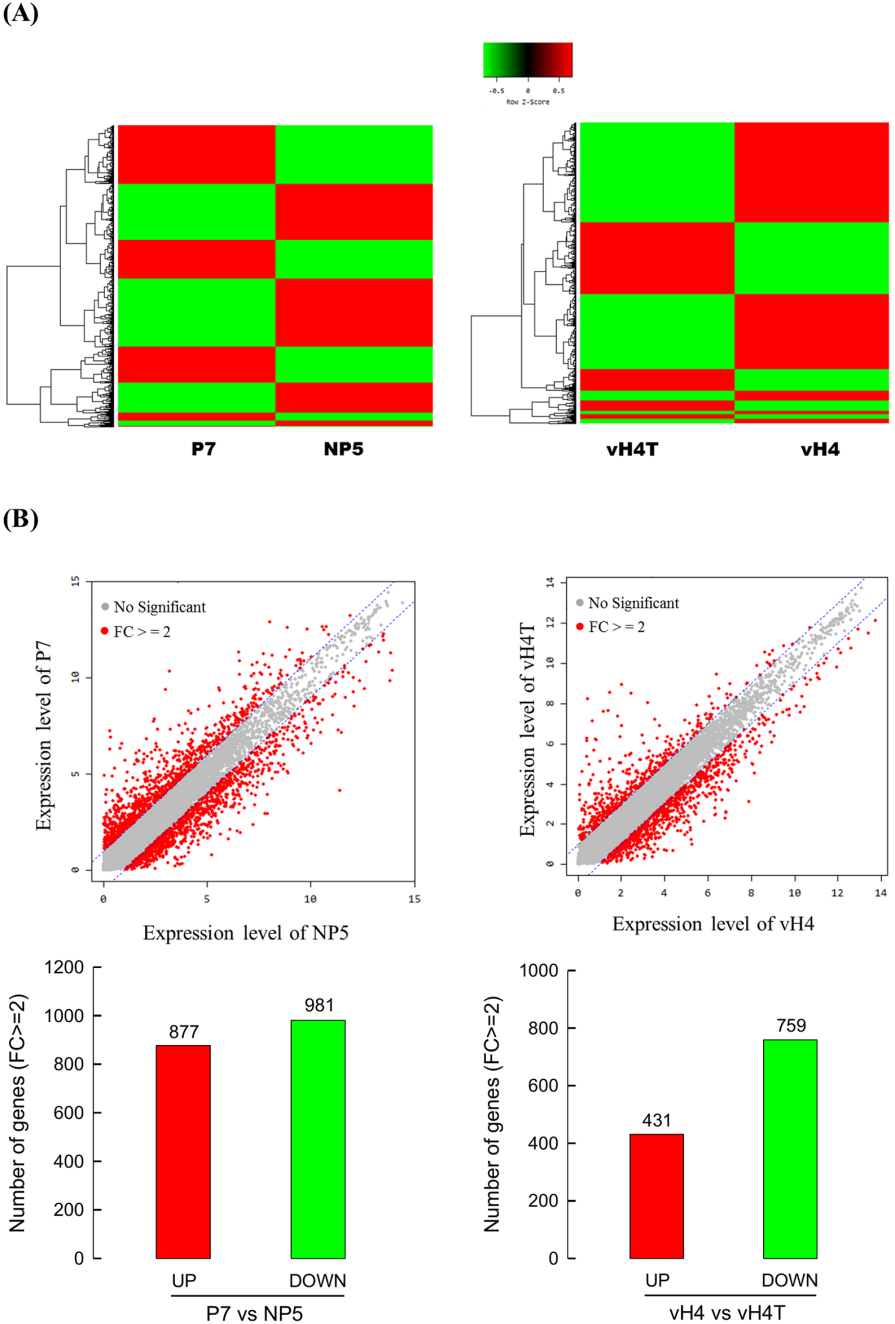
Alteration of host gene expression by parasitism of *C*. *plutellae* or expression of a viral gene *CpBV-H4*. To determine parasitic control of *P*. *xylostella* gene expression, transcripts were subjected to RNA-Seq from 4^th^ instar larvae parasitized (‘P7’) or nonparasitized (‘NP5’) by *C*. *plutellae*. To determine CpBV-H4 control of host gene expression, transcripts were subjected to RNA-Seq from 4^th^ instar larvae transiently expressing CpBV-H4 containing N-terminal tail (‘vH4’) or truncated CpBV-H4 (‘vH4T’) after deleting N-terminal tail. (A) Heat map analysis of expression patterns in four different treatments. (B) DEG analysis of transcripts in their FPKM levels between NP5 and P7 or between vH4 and vH4T. Red spots indicate transcripts exhibiting more than two-fold change (‘FC’) in expression level. ‘UP’ and ‘DOWN’ represent up-regulation and down-regulation of gene expression, respectively, in the two DEG groups.

*C*. *plutellae* alters host gene expression via parasitic factors including CpBV-H4 which has been regarded as a regulator of host gene expression at transcriptional level by an epigenetic mode [14]. To determine genes regulated only by CpBV-H4, four RNA-Seq data (S1 Table) were compared in fragment per kilobase of transcript per million mapped reads (FPKM) levels: nonparasitized larvae (‘NP5’), parasitized larvae (‘P7’), nonparasitized larvae transiently expressing *CpBV-H4* (‘vH4’), and truncated *CpBV-H4* (‘vH4T’). All four transcriptomes had reads of more than 60 Gb in size. More than 50% of these sequences were mapped to *P*. *xylostella* genome sequence with P7 having lower mapping rate (S1 Fig). Relatively low mapping ratio of P7 might be due to contaminants of immature *C*. *plutellae* wasp and teratocyte transcripts because all reads were mapped only to *P*. *xylostella* genome. Using these four transcriptomes, differentially expressed gene (DEG) analyses were performed between NP5 and P7 to obtain specific host genes regulated by parasitism or between vH4 and vH4T to search for specific host genes regulated only by *CpBV-H4* expression (Fig 1). Expression pattern analysis showed that the number of major clades in the comparison between P7 and NP5 was higher than that between vH4 and vH4T (Fig 1A). DEG analysis between P7 and NP5 showed that 1,858 host genes exhibited more than 2-fold of changes in expression level after parasitism, including 877 up-regulated genes and 981 down-regulated genes (Fig 1B). It also showed that 1,190 host genes exhibited more than 2-fold of changes in expression level after *CpBV-H4* expression by comparing the transcript levels between vH4 and vH4T treatments, including 431 up-regulated host genes and 759 down-regulated host genes.

**Fig 2:**
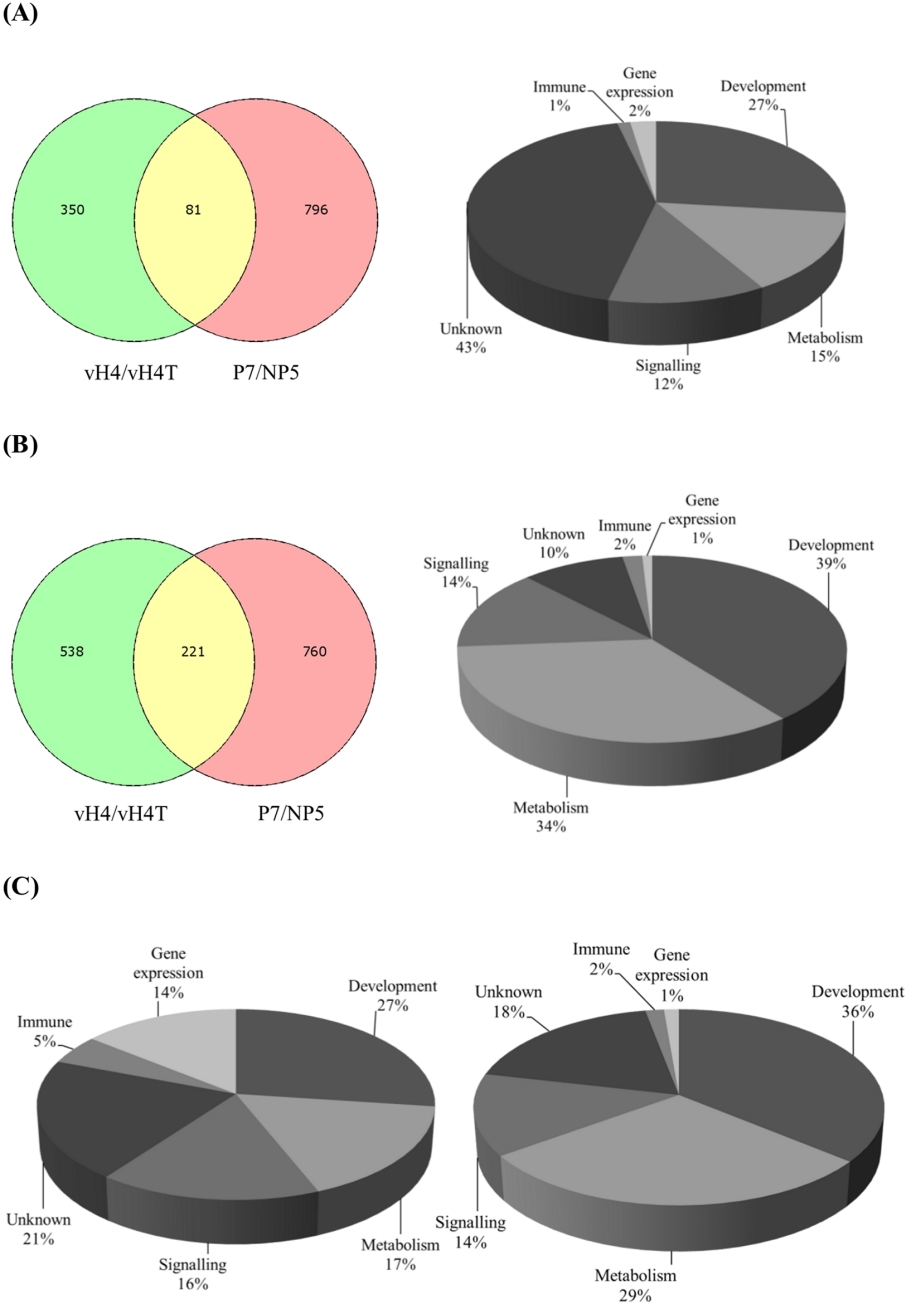
Influence of CpBV-H4 on altering host gene expression of *P*. *xylostella* during *C*. *plutellae* parasitism. (A) Genes up-regulated by both parasitism and CpBV-H4 (left panel). Gene ontology (GO) analysis of 81 commonly up-regulated genes (right panel). (B) Genes down-regulated by both parasitism and CpBV-H4 (left panel). GO analysis of 221 commonly down-regulated genes (right panel). ‘vH4/vH4T’ represents specific genes regulated by CpBV-H4 (vH4) by deducting background genes induced by the truncated CpBV-H4 (vH4T). ‘P7/NP5’ represents specific genes regulated by parasitism by deducting background genes expressing under nonparasitized status. (C) GO analysis of total host genes regulated by parasitism (left panel) and specific host genes regulated by CpBV-H4 (right panel). Host genes were identified from *P*. *xylostella* genome database (http://iae.fafu.edu.cn/DBM/index.php) and annotated with GO program (https://en.wikipedia.org/wiki/Gene_ontology).

To determine the influence of CpBV-H4 on the regulation of host genes during *C*. *plutellae* parasitism, parasitism-specific DEG (‘P7/NP5’, 1,858 genes) and CpBV-H4 DEG (‘vH4/vH4T’, 1,190 genes) were compared (Fig 2). This comparison resulted in 302 genes overlapped between the two DEGs, including 81 up-regulated genes (Fig 2A and Table 1) and 221 down-regulated genes (Fig 2B and Table 2). Almost half of these genes up-regulated by CpBV-H4 had no functional annotation whereas almost 70% genes down-regulated by CpBV-H4 were predicted to have functions in development and metabolism. These DEG analyses indicated that *CpBV-H4* expression contributed 16.3% (302/1,858 x 100) to *C*. *plutellae* parasitism with respect to host gene regulation. In addition, CpBV-H4 appeared to control specific target genes because host genes regulated by CpBV-H4 were significantly (X^2^ = 1,804.12; df = 10; *P* < 0.0001) different in functional categories compared to those regulated by *C*. *plutellae* parasitism (Fig 2C).

**Table 1:** List of 81 up-regulated host genes after expressing viral gene *CpBV-H4* in *P*. *xylostella*. Gene expression levels were compared between late third instar larvae parasitized (P7) or nonparasitized (NP5) by *C*. *plutellae* and expressed in fold change (FC) with respect to FPKM values. Gene identification (ID) followed the annotation of *P*. *xylostella* Genome Database (http://iae.fafu.edu.cn/DBM/index.php).

Genes	Gene ID	FC (P7/NP5)	Functional categories
Larval cuticle protein LCP-30	Px001339	63.3	Development
Larval cuticle protein LCP-30	Px016045	63.3	Development
Death-associated small cytoplasmic leucine-rich protein	Px012314	40.4	Development
Urbain	Px010057	29.6	Signaling
Retinol dehydrogenase	Px017386	25.4	Metabolism
Collagen alpha chain	Px013919	21.8	Development
Hypothetical	Px000870	16.6	Unknown
Neurofilament heavy polypeptide	Px001530	15.8	Signaling
Hypothetical	Px010443	15.8	Unknown
Sulfotransferase	Px000114	15.1	Metabolism
Methyltransferase-like protein	Px002962	13.4	Development
Hypothetical	Px015205	12.0	Unknown
Putative uncharacterized protein	Px014022	7.6	Unknown
Hypothetical	Px009883	7.5	Unknown
Protein lethal essential for life	Px003977	7.5	Development
Hypothetical	Px012012	7.1	Unknown
Hypothetical	Px011355	6.9	Unknown
Hypothetical	Px017694	6.5	Unknown
Protein PF14_0175	Px008075	5.4	Signaling
Putative cuticle protein	Px005453	5.3	Development
Neurofilament heavy polypeptide	Px017104	5.2	Signaling
Hypothetical	Px003919	5.0	Unknown
Alpha-tocopherol transfer protein-like	Px004656	4.8	Development
Hypothetical	Px006831	4.7	Unknown
LIX1-like protein	Px013516	4.4	Development
Hypothetical	Px008280	4.4	Unknown
Hypothetical	Px003203	4.3	Unknown
Hypothetical	Px015340	4.2	Unknown
Adenosine deaminase	Px004490	4.1	Metabolism
Gustatory receptor candidate	Px008611	3.9	Signaling
Scavenger receptor class B member 1	Px008150	3.8	Signaling
Protein lethal essential for life	Px012769	3.8	Development
Glutathione S-transferase	Px006105	3.7	Metabolism
Unknown function	Px002808	3.6	Unknown
Putative cuticle protein	Px014258	3.4	Development
Serpin	Px015089	3.4	Immune
Hypothetical	Px010540	3.4	Unknown
Hypothetical	Px013167	3.4	Unknown
Putative inorganic phosphate cotransporter	Px012902	3.4	Metabolism
Fibrohexamerin	Px001085	3.4	Metabolism
Putative inorganic phosphate cotransporter	Px016965	3.3	Metabolism
Angiotensin-converting enzyme	Px012642	3.3	Metabolism
Hypothetical	Px010625	3.3	Unknown
Neurofilament heavy polypeptide	Px016161	3.1	Signaling
Clavesin-1	Px012514	3.1	Development
Hypothetical	Px008793	3.1	Unknown
Hypothetical	Px002698	3.0	Unknown
Decaprenyl-diphosphate synthase	Px008402	3.0	Metabolism
Ejaculatory bulb-specific protein	Px009195	3.0	Development
Osiris	Px010922	2.9	Development
Putative uncharacterized protein	Px007085	2.9	Unknown
Neprilysin	Px017926	2.9	Gene expression
Probable nuclear hormone receptor	Px008400	2.9	Signaling
Hypothetical	Px004742	2.7	Unknown
Putative uncharacterized protein	Px009680	2.7	Unknown
Hypothetical	Px001678	2.7	Unknown
Proteasome subunit alpha type-2	Px010913	2.7	Development
Hypothetical	Px001472	2.7	Unknown
Hypothetical	Px012390	2.7	Unknown
Helix-loop-helix protein Delilah	Px005488	2.6	Development
Putative cuticle protein CPH36	Px015917	2.5	Development
Clavesin-1	Px004516	2.5	Development
Hypothetical	Px003928	2.5	Unknown
Probable dolichol-phosphate mannosyltransferase	Px012743	2.4	Metabolism
Hypothetical	Px006833	2.4	Unknown
Hypothetical	Px008161	2.4	Unknown
Hypothetical	Px007157	2.4	Unknown
Hypothetical	Px006388	2.3	Unknown
Hypothetical	Px008548	2.3	Unknown
Putative odorant-binding protein A10	Px014885	2.3	Signaling
Hypothetical	Px012010	2.2	Unknown
Putative inorganic phosphate cotransporter	Px013082	2.2	Metabolism
Alpha-tocopherol transfer protein-like	Px001573	2.2	Development
Carboxypeptidase B	Px005373	2.2	Development
Hypothetical	Px010142	2.2	Unknown
Endochitinase	Px008062	2.2	Development
Hypothetical	Px003497	2.2	Unknown

**Table 2:** List of 221 host genes down-regulated by the expression of a viral gene *CpBV-H4* in *P*. *xylostella*. Gene expression levels were compared between late third instar larvae parasitized (P7) or nonparasitized (NP5) by *C*. *plutellae* and expressed in fold change (FC) with respect to FPKM values. Gene identification (ID) followed the annotation of *P*. *xylostella* Genome Database (http://iae.fafu.edu.cn/DBM/index.php). Negative value indicates decreased expression of P7 compared to NP5.

Genes	Gene ID	FC (P7/NP5)	Functional categories
Lipase	Px011477	-2.0	Metabolism
Cytosolic β-glucosidase	Px010022	-2.0	Metabolism
Trypsin	Px007677	-2.0	Development
Trypsin	Px006570	-2.0	Development
Superoxide dismutase [Cu-Zn]	Px001161	-2.1	Signaling
Chitin deacetylase	Px001430	-2.1	Development
Esterase	Px011443	-2.1	Metabolism
Mitochondrial 2-oxoglutarate	Px009263	-2.1	Metabolism
Sodium/potassium/calcium exchanger	Px002564	-2.1	Metabolism
Trypsin	Px005242	-2.1	Development
Cytochrome b5-related protein	Px010558	-2.1	Development
Beta-ureidopropionase	Px017763	-2.1	Signaling
Glutathione S-transferase	Px006481	-2.1	Metabolism
GTP: AMP phosphotransferase, mitochondrial	Px012467	-2.1	Metabolism
Cytochrome b-c1 complex	Px011320	-2.1	Metabolism
Inositol oxygenase	Px016791	-2.1	Signaling
Trypsin	Px007619	-2.1	Development
Trypsin	Px016058	-2.1	Development
Aminoacylase	Px001371	-2.1	Metabolism
Protein henna similar to PH4H_DROME	Px004945	-2.1	Signaling
Labial Similar to D6W945_TRICA	Px016748	-2.2	Signaling
Hexokinase	Px010425	-2.2	Metabolism
General odorant-binding protein	Px004433	-2.2	Signaling
Hexokinase	Px003540	-2.2	Metabolism
Serine protease	Px016741	-2.2	Immune
Lipase	Px012694	-2.2	Metabolism
Monocarboxylate transporter	Px006257	-2.2	Signaling
Aminopeptidase N	Px001708	-2.2	Metabolism
Peritrophin type-A domain protein	Px004696	-2.2	Metabolism
Chymotrypsin	Px005554	-2.2	Development
Hypothetical	Px014003	-2.2	Unknown
1-acyl-sn-glycerol-3-phosphate acyltransferase-α	Px017143	-2.2	Metabolism
Collagenase	Px005340	-2.2	Development
Acyl-CoA oxidase	Px017102	-2.2	Metabolism
SWI/SNF complex subunit SMARCC2	Px016803	.2.2	Immune
Hypothetical	Px014974	-2.3	Unknown
Lipase	Px012695	-2.3	Metabolism
Uridine phosphorylase	Px009019	-2.3	Metabolism
Luciferin 4-monooxygenase	Px010831	-2.3	Metabolism
Arylsulfatase B	Px010550	-2.3	Development
4-coumarate—CoA ligase	Px011113	-2.3	Metabolism
3-oxoacyl-[acyl-carrier-protein] reductase FabG	Px018005	-2.3	Metabolism
Cytochrome b561 domain-containing protein	Px008446	-2.3	Development
Protein 5NUC	Px012027	-2.3	Signaling
Sensory neuron membrane protein	Px013824	-2.4	Signaling
Collagenase	Px012582	-2.4	Development
Chymotrypsin BI	Px007900	-2.4	Development
Aminoacylase-1A	Px008322	-2.4	Metabolism
Long-chain-fatty-acid—CoA ligase ACSBG2	Px008837	-2.4	Metabolism
Membrane alanyl aminopeptidase	Px008278	-2.4	Signaling
Pancreatic triacylglycerol lipase	Px002296	-2.4	Metabolism
Peritrophin type-A domain protein 2	Px007019	-2.4	Metabolism
Chymotrypsin-like elastase family member 2B	Px011888	-2.4	Development
Protein ETHE1, mitochondrial	Px005329	-2.5	Metabolism
Hexokinase	Px001060	-2.5	Metabolism
Putative inorganic phosphate cotransporter	Px014024	-2.5	Metabolism
Zinc carboxypeptidase A	Px000994	-2.5	Metabolism
Glucose dehydrogenase	Px011825	-2.5	Metabolism
Solute carrier family 22 member 21	Px017195	-2.5	Signaling
Ribosome-binding protein	Px009784	-2.5	Gene expression
Myrosinase	Px002081	-2.5	Metabolism
Proton-coupled amino acid transporter	Px007090	-2.5	Signaling
Probable E3 ubiquitin-protein ligase sinah	Px008209	-2.5	Signaling
Trypsin, alkaline B	Px009284	-2.5	Signaling
Transferrin	Px011514	-2.6	Signaling
Retinoid-inducible serine carboxypeptidase	Px000211	-2.6	Development
Membrane alanyl aminopeptidase	Px003754	-2.6	Signaling
Carboxypeptidase O	Px000991	-2.6	Development
Phosphotriesterase-related protein	Px009117	-2.6	Metabolism
Trypsin	Px016057	-2.6	Development
Hypothetical	Px010793	-2.6	Unknown
Hypothetical	Px008726	-2.7	Unknown
Facilitated trehalose transporter	Px009445	-2.7	Development
Glutathione S-transferase	Px010078	-2.7	Metabolism
Putative inorganic phosphate cotransporter	Px013678	-2.7	Development
Sedoheptulokinase	Px007641	-2.7	Development
Acyl-CoA synthetase family member	Px016733	-2.7	Metabolism
Hypothetical	Px004934	-2.7	Unknown
Prostaglandin reductase	Px009370	-2.8	Immune
Membrane alanyl aminopeptidase	Px008277	-2.8	Metabolism
Hypothetical	Px013342	-2.8	Unknown
Plasma glutamate carboxypeptidase	Px009761	-2.8	Metabolism
Transferrin	Px012137	-2.8	Signaling
2-Oxoisovalerate dehydrogenase subunit alpha	Px006816	-2.8	Metabolism
Glyoxylate reductase/hydroxypyruvate reductase	Px004525	-2.8	Metabolism
Inactive dipeptidyl peptidase	Px013212	-2.8	Metabolism
Facilitated trehalose transporter	Px012298	-2.8	Development
Trypsin	Px001804	-2.9	Development
Monocarboxylate transporter	Px005246	-2.9	Signaling
Sucrose-6-phosphate hydrolase	Px001761	-2.9	Metabolism
Lipase	Px005804	-2.9	Metabolism
Galactokinase	Px005810	-2.9	Metabolism
Multiple C2 and transmembrane domain	Px005022	-2.9	Signaling
C-1-tetrahydrofolate synthase, cytoplasmic	Px003316	-2.9	Development
Lysine-specific demethylase NO66	Px009382	-3.0	Immune
Gamma-glutamyl hydrolase A	Px011489	-3.0	Metabolism
Cytochrome P450 6B6	Px005902	-3.0	Development
Collagenase	Px007902	-3.1	Development
Midgut protein Lsti99	Px014861	-3.1	Development
Hypothetical	Px005937	-3.1	Unknown
Arylphorin subunit alpha	Px007028	-3.1	Gene expression
Trypsin	Px005241	-3.1	Development
Membrane alanyl aminopeptidase	Px003755	-3.1	Signaling
GH11122	Px012786	-3.1	Development
Luciferin 4-monooxygenase	Px016655	-3.1	Metabolism
Hypothetical	Px014245	-3.1	Unknown
Apolipophorins	Px015730	-3.1	Development
Ribose-phosphate pyrophosphokinase	Px011035	-3.1	Metabolism
Chymotrypsin-C	Px007676	-3.2	Development
Xanthine dehydrogenase	Px002720	-3.3	Metabolism
Facilitated trehalose transporter	Px004014	-3.3	Development
Myrosinase	Px009427	-3.3	Metabolism
Retinol dehydrogenase	Px000793	-3.3	Metabolism
ACYPI004563 protein	Px016078	-3.4	Signaling
Probable peroxisomal acyl-coenzyme A oxidase 1	Px001531	-3.4	Metabolism
Collagenase	Px005342	-3.4	Development
Hypothetical	Px012051	-3.4	Unknown
Trypsin	Px007621	-3.4	Development
Transmembrane inner ear expressed protein	Px007550	-3.4	Signaling
Probable dihydropyrimidine dehyd. [NADP+]	Px014464	-3.5	Metabolism
Trypsin	Px015277	-3.5	Development
L-ascorbate oxidase	Px001443	-3.5	Metabolism
3-oxoacyl-[acyl-carrier-protein] reductase FabG	Px016235	-3.5	Metabolism
Acetylcholinesterase	Px011049	-3.5	Development
Estradiol 17-β-dehydrogenase	Px015642	-3.6	Development
galactose-1-phosphate uridylyltransferase	Px004395	-3.6	Development
Dihydropyrimidine dehydrogenase [NADP+]	Px003428	-3.6	Development
Esterase	Px006430	-3.6	Development
Angiotensin-converting enzyme	Px012643	-3.6	Development
Aminomethyltransferase, mitochondrial	Px011054	-3.7	Metabolism
Ecdysteroid UDP-glucosyltransferase	Px000872	-3.7	Development
Sodium- and chloride-dep. glycine transporter	Px009514	-3.8	Signaling
Dihydropyrimidine dehydrogenase [NADP+]	Px000264	-3.8	Development
Hypothetical	Px004235	-3.8	Unknown
Angiotensin-converting enzyme	Px001633	-3.8	Signaling
Hypothetical	Px001337	-3.9	Unknown
Hypothetical	Px010854	-3.9	Unknown
Esterase	Px002735	-3.9	Metabolism
Phosphoenolpyruvate carboxykinase [GTP]	Px015376	-3.9	Metabolism
Mitochondrial ornithine transporter 1	Px007072	-3.9	Metabolism
Luciferin 4-monooxygenase	Px003138	-3.9	Metabolism
Ecdysteroid UDP-glucosyltransferase	Px004854	-3.9	Development
Trypsin	Px006572	-4.0	Development
Lipase	Px011610	-4.0	Development
Peritrophic matrix insect intestinal mucin	Px007895	-4.1	Development
Glucosidase KIAA1161	Px002046	-4.1	Metabolism
Acetylcholinesterase	Px000089	-4.1	Development
Luciferin 4-monooxygenase	Px012806	-4.1	Development
Estradiol 17-β-dehydrogenase	Px008771	-4.1	Development
Cytochrome P450 6B5	Px013454	-4.1	Signaling
Trypsin	Px002864	-4.2	Development
Bifunctional purine biosynthesis protein	Px011885	-4.3	Metabolism
Carboxypeptidase	Px000996	-4.3	Development
Trypsin	Px000107	-4.4	Development
Phosphoserine phosphatase	Px000750	-4.4	Development
Hypothetical	Px012836	-4.5	Unknown
C-1-tetrahydrofolate synthase, cytoplasmic	Px007305	-4.5	Metabolism
Isovaleryl-CoA dehydrogenase, mitochondrial	Px014703	-4.6	Metabolism
Pancreatic lipase-related protein	Px005193	-4.7	Development
Sorbitol dehydrogenase	Px000215	-4.8	Metabolism
Probable maltase	Px003486	-4.8	Development
Multifunctional protein ADE2	Px011706	-4.9	Signaling
Phosphoserine phosphatase	Px007835	-4.9	Development
Acetylcholinesterase	Px009940	-4.9	Development
Trypsin	Px012568	-5.0	Development
Hypothetical	Px006985	-5.2	Unknown
Sorbitol dehydrogenase	Px004996	-5.3	Metabolism
Juvenile hormone esterase	Px012592	-5.3	Development
Synaptic vesicle glycoprotein	Px001753	-5.3	Signaling
Hypothetical	Px004933	-5.4	Unknown
Myrosinase 1	Px006942	-5.4	Metabolism
Pancreatic triacylglycerol lipase	Px000644	-5.5	Development
Esterase	Px011756	-5.6	Development
Ecdysteroid-regulated protein	Px009634	-5.6	Development
Glutaryl-CoA dehydrogenase, mitochondrial	Px011286	-5.7	Development
Peritrophin-1	Px010130	-5.8	Signaling
Elongation of very long chain fatty acids protein	Px013748	-5.9	Development
Larval cuticle protein	Px003260	-6.0	Development
Peritrophic matrix insect intestinal mucin	Px007897	-6.1	Development
Ecdysteroid-regulated protein	Px011111	-6.1	Development
Antennal esterase	Px011755	-6.1	Development
Phosphoenolpyruvate carboxykinase	Px010887	-6.1	Metabolism
Pancreatic triacylglycerol lipase	Px012011	-6.2	Development
Hypothetical	Px013960	-6.4	Unknown
Glycine N-methyltransferase	Px003291	-6.4	Immune
Sialin	Px004317	-6.5	Development
Myrosinase 1	Px006054	-6.6	Development
Oxidoreductase ucpA	Px002029	-6.7	Development
Hypothetical	Px001077	-6.7	Unknown
Prostaglandin reductase	Px006403	-6.9	Immune
Trypsin	Px010386	-7.0	Development
Choline dehydrogenase, mitochondrial	Px002926	-7.1	Metabolism
Facilitated trehalose transporter	Px002482	-7.1	Development
Putative acyl-CoA-binding protein	Px000524	-7.2	Metabolism
Sucrose-6-phosphate hydrolase	Px004218	-7.2	Development
Luciferin 4-monooxygenase	Px016492	-7.3	Metabolism
Phosphoenolpyruvate carboxykinase	Px015377	-7.3	Development
Zinc carboxypeptidase A	Px015831	-7.4	Metabolism
Putative acyl-CoA-binding protein	Px001605	-7.4	Metabolism
Adenylate kinase isoenzyme 1	Px018022	-7.4	Metabolism
Hypothetical	Px012853	-7.5	Unknown
Carboxypeptidase B	Px010017	-7.5	Development
Probable D-xylulose reductase A	Px013954	-7.7	Development
Collagenase	Px013665	-7.7	Development
Alpha-amylase 4N	Px000395	-8.0	Metabolism
Larval cuticle protein 16/17	Px003261	-8.0	Development
Esterase	Px011757	-8.5	Development
Hypothetic	Px006547	-8.5	Unknown
Carboxypeptidase	Px015830	-8.7	Development
Collagenase	Px005341	-8.8	Development
Glutathione S-transferase	Px006106	-8.9	Signaling
Lactase-phlorizin hydrolase	Px005277	-9.1	Metabolism
Fibrohexamerin	Px001076	-10.2	Signaling
Glucose dehydrogenase [acceptor]	Px008505	-10.2	Development
Putative acyl-CoA-binding protein	Px017039	-11.8	Metabolism
Hypothetical	Px013308	-11.9	Unknown
α-amylase	Px000394	-13.2	Metabolism
Chitin binding PM protein	Px001431	-13.8	Development
Trypsin	Px012570	-14.6	Development
Lactase-phlorizin hydrolase	Px011160	-15.2	Development
Repetitive proline-rich cell wall protein	Px010466	-16.7	Metabolism
Vesicular glutamate transporter	Px014303	-21.5	Signaling
Hypothetical	Px016820	-153.5	Unknown

### Sequence specificity of CpBV-H4-joining sites on host chromosomes

**Fig 3:**
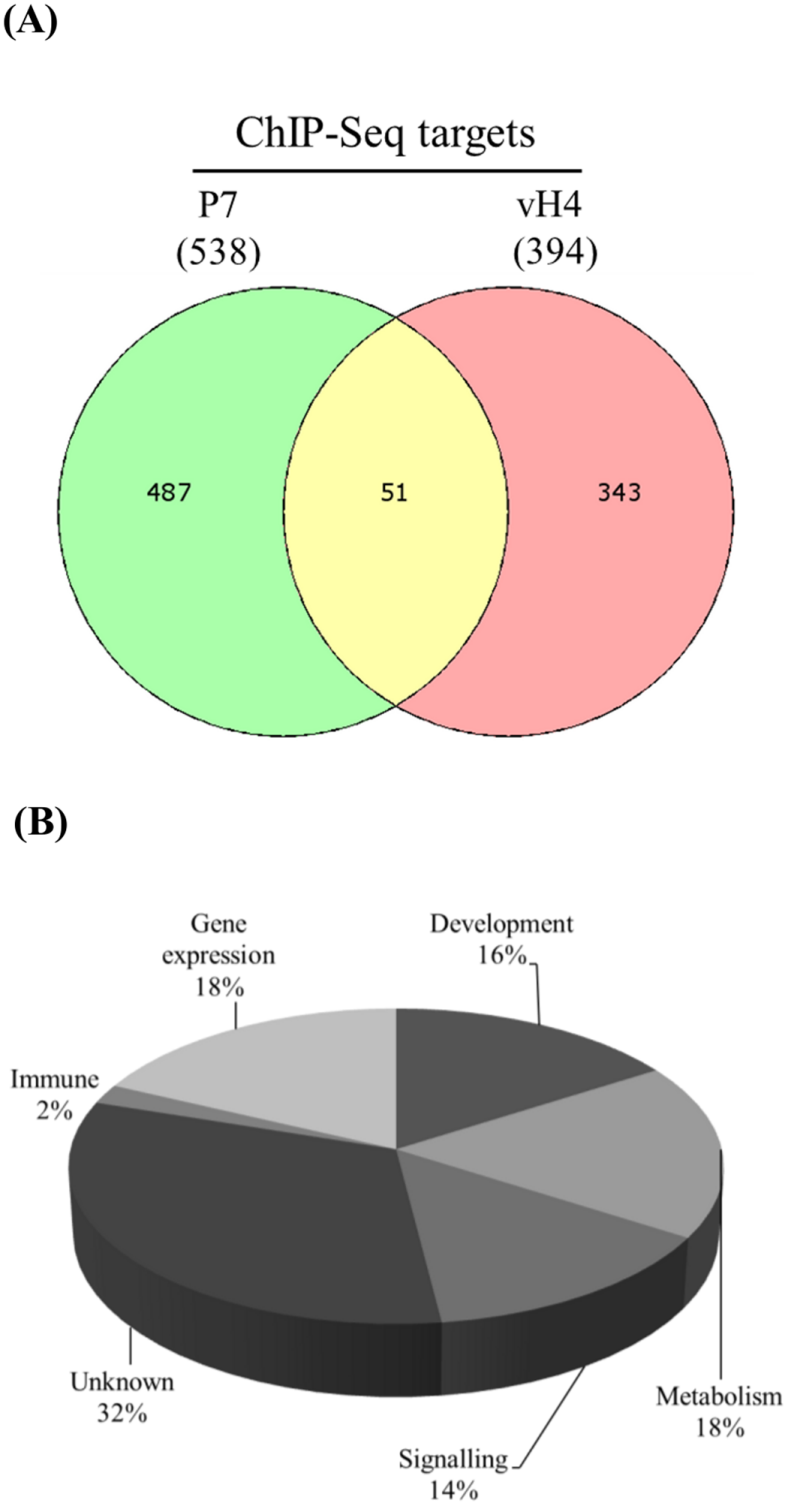
Total joining sites of CpBV-H4 on *P*. *xylostella* genome by ChIP-Seq analysis. ChIP-Seq with a polyclonal antibody specific to CpBV-H4 was performed against chromatins extracted from host larvae parasitized by *C*. *plutellae* (‘P7’) or from host larvae transiently expressing CpBV-H4 (‘vH4’). (A) A total of 538 joining sites of CpBV-H4 on chromosomes, of which 51 sites were also targets of ChIP against vH4-treated larvae. (B) GO analysis of the 51 ChIP target genes.

To determine the specificity of CpBV-H4-joining sites on host chromosomes, ChIP-Seq analysis was performed using a polyclonal antibody raised against CpBV-H4. ChIP isolated 1,498 targets against chromatins originated from larvae at late parasitism (‘P7’). After deleting nonspecific sites detected from ChIP-Seq analysis using chromatins originated from NP5 larvae, P7-specific ChIP targets had 538 sites (Fig 3A). Similar ChIP-Seq was performed against chromatins extracted from larvae transiently expressing *CpBV-H4* and 394 targets were obtained after deleting nonspecific sites detected from ChIP-Seq analysis using larvae expressing truncated *CpBV-H4*. There were 51 common core target genes (‘vH4-ChIP1 ~ vH4-ChIP51’) between P7-specific ChIP targets and CpBV-H4-specific ChIP targets (Table 3). Genes close (within 1 kb) to these 51 core target sites were predicted to have functions in development, metabolism, immunity, signaling, and gene expression (Fig 3B).

**Table 3:** List of 51 common ChIP targets of CpBV-H4 (vH4) on *P*. *xylostella* genome from analysis of chromatin immunoprecipitation followed by deep sequencing (ChIP-Seq).

ChIP targets	Location on *P*. *xylostella* genome	Distance (bp) to nearest CDS^a^	Nearest CDS (GenBank accession #)	FC (P7/NP5) ^b^	Functional categories
vH4-ChIP-1	ChrLG-1 (267416–267973)	-557	Hypothetical (Px013825)	5.0	Unknown
vH4-ChIP-2	ChrLG-5 (276105–276260)	155	Hypothetical (Px008241)	4.2	Unknown
vH4-ChIP-3	ChrLG-5 (215111–219713)	-602	Cleft lip and palate transmembrane protein 1-like protein (Px008237)	-10.3	Signaling
vH4-ChIP-4	ChrLG-5 (316488–318790)	-473	Centrosomal protein of 97 kDa (Px004309)	-3.0	Development
vH4-ChIP-5	ChrLG-6 (478831–486427)	596	Uncharacterized protein KIAA0090 homolog (Px003642)	10.8	Unknown
vH4-ChIP-6	ChrLG-7 (156448–162473)	-447	Zinc finger protein (Px011497)	12.2	Signaling
vH4-ChIP-7	ChrLG-12 (239288–249465)	472	Mitochondrial glutamate carrier (Px008056)	-14.2	Metabolism
vH4-ChIP-8	ChrLG-12 (59259–80699)	683	GJ16239 (Px008047)	8.5	Metabolism
vH4-ChIP-9	ChrLG-14 (271091–271922)	831	Putative uncharacterized protein (Px010174)	-10.1	Unknown
vH4-ChIP-10	ChrLG-17 (1121617–1126344)	-526	Protein penguin (Px009354)	8.0	Development
vH4-ChIP-11	ChrLG-21 (882018–882712)	694	HIG1 domain family member 2A (Px002719)	-4.2	Gene expression
vH4-ChIP-12	ChrLG-22 (883663–922749)	469	Polycomb protein eed-A (Px002998	18.2	Development
vH4-ChIP-13	ChrLG-22 (1306408–1317112)	-704	Uncharacterized protein KIAA1370 (Px003709)	-2.3	Unknown
vH4-ChIP-14	ChrLG-24 (470439–478675)	236	Endothelial zinc finger protein induced by TNF alpha (Px006727)	5.0	Development
vH4-ChIP-15	ChrLG-26 (50771–68538)	767	Zinc finger protein (Px010512	25.1	Signaling
vH4-ChIP-16	ChrLG-UN (16658–37064)	406	Leucine-rich repeat-containing protein (Px016400)	-7.5	Metabolism
vH4-ChIP-17	ChrLG-UN (107271–120081)	810	Aldehyde dehydrogenase family 1 member L1 (Px013663)	-45.6	Metabolism
vH4-ChIP-18	ChrLG-UN (204710–223658)	684	SID1 transmembrane family member 1 (Px017204)	-40.4	Signaling
vH4-ChIP-19	ChrLG-UN (75155–82548)	-582	GTPase-activating protein (Px014828)	-25.0	Metabolism
vH4-ChIP-20	ChrLG-UN (12872–14358)	829	Vacuolar protein sorting-associated protein (Px001552)	24.0	Metabolism
vH4-ChIP-21	ChrLG-UN (224911–226167)	-738	α-(1,3)-fucosyltransferase (Px005358)	18.2	Metabolism
vH4-ChIP-22	ChrLG-UN (3739–5950)	621	Mitochondrial intermediate peptidase (Px002154)	18.2	Metabolism
vH4-ChIP-23	ChrLG-UN (1709593–1710105)	512	Hypothetical (Px005915)	-16.9	Unknown
vH4-ChIP-24	ChrLG-UN (696255–714576)	944	Dual serine/threonine and tyrosine protein kinase (Px006800)	-16.0	Immune
vH4-ChIP-25	ChrLG-UN (44835–47452)	-654	Hypothetical (Px005285)	-16	Unknown
vH4-ChIP-26	ChrLG-UN (4769–6486)	26	Putative transposase ykgN (Px017967)	-14.2	Gene expression
vH4-ChIP-27	ChrLG-UN (88684–107215)	828	Hypothetical (Px013605)	14.2	Unknown
vH4-ChIP-28	ChrLG-UN (172060–172677)	-617	Hypothetical (Px005697)	-13.7	Unknown
vH4-ChIP-29	ChrLG-UN (998583–1039637)	125	Nucleolar MIF4G domain-containing protein 1 homolog (Px014314)	-13.4	Gene expression
vH4-ChIP-30	ChrLG-UN (78554–80038)	-832	MORN repeat-containing protein (Px014423)	13.1	Gene expression
vH4-ChIP-31	ChrLG-UN (186099–186577)	478	Hypothetical (Px012337)	13.0	Unknown
vH4-ChIP-32	ChrLG-UN (475306–475995)	-689	A7S037_NEMVE; Predicted protein (Px004214)	-12.3	Gene expression
vH4-ChIP-33	ChrLG-UN (13224–59610)	386	Rapamycin-insensitive companion of m-Tor (Px015368)	12.1	Signaling
vH4-ChIP-34	ChrLG-UN (112006–130292)	286	Exostosin-2 (Px013363)	12.0	Gene expression
vH4-ChIP-35	ChrLG-UN (16056–17294)	755	Facilitated trehalose transporter (Px016880)	-10.4	Development
vH4-ChIP-36	ChrLG-UN (1622315–1627191)	876	Regulator of G-protein signaling (Px016347)	-10.3	Signaling
vH4-ChIP-37	ChrLG-UN (1345193–1351114)	-921	Ras-related protein RabJ (Px006237)	-10.1	Signaling
vH4-ChIP-38	ChrLG-UN (600031–600513)	-482	Probable serine hydrolase (Px008214)	8.6	Metabolism
vH4-ChIP-39	ChrLG-UN (89183–118306)	-987	Protein PAT1 homolog (Px010075)	-8.4	Gene expression
vH4-ChIP-40	ChrLG-UN (273969–274525)	556	Hypothetical (Px007509)	7.45	Unknown
vH4-ChIP-41	ChrLG-UN (108141–109100)	959	Hypothetical (Px009739)	-6.8	Unknown
vH4-ChIP-42	ChrLG-UN (2292630–2293702)	-261	Larval cuticle protein LCP-30 (Px003250)	-6.6	Development
vH4-ChIP-43	ChrLG-UN (2294845–2297228)	118	Putative cuticle protein (Px003251)	-6.6	Development
vH4-ChIP-44	ChrLG-UN (448023–448313)	290	Hypothetical (Px004665)	6.5	Unknown
vH4-ChIP-45	ChrLG-UN (709982–756563)	122	Protein dpy-19 homolog (Px002457)	6.4	Metabolism
vH4-ChIP-46	ChrLG-UN (1513940–1538960)	543	UBX domain-containing protein 4 (Px006242)	5.8	Gene expression
vH4-ChIP-47	ChrLG-UN (192843–194073)	-891	Hypothetical (Px012789)	-10.7	Unknown
vH4-ChIP-48	ChrLG-UN (390910–391918)	312	Hypothetical (Px003836)	-7.7	Unknown
vH4-ChIP-49	ChrLG-UN (417484–423132)	648	Hypothetical (Px004564)	-6.5	Unknown
vH4-ChIP-50	ChrLG-UN (288781–289959)	669	Probable 39S ribosomal protein L45 (Px005650)	6.3	Gene expression
vH4-ChIP-51	ChrLG-UN (1681979–1682541)	-562	Trypsin (Px002590)	-4.9	Development

^a^ + and - represent upstream and downstream, respectively, from the nearest coding DNA sequence (CDS)

^b^ Fold change (FC) in FPKM values

**Fig 4:**
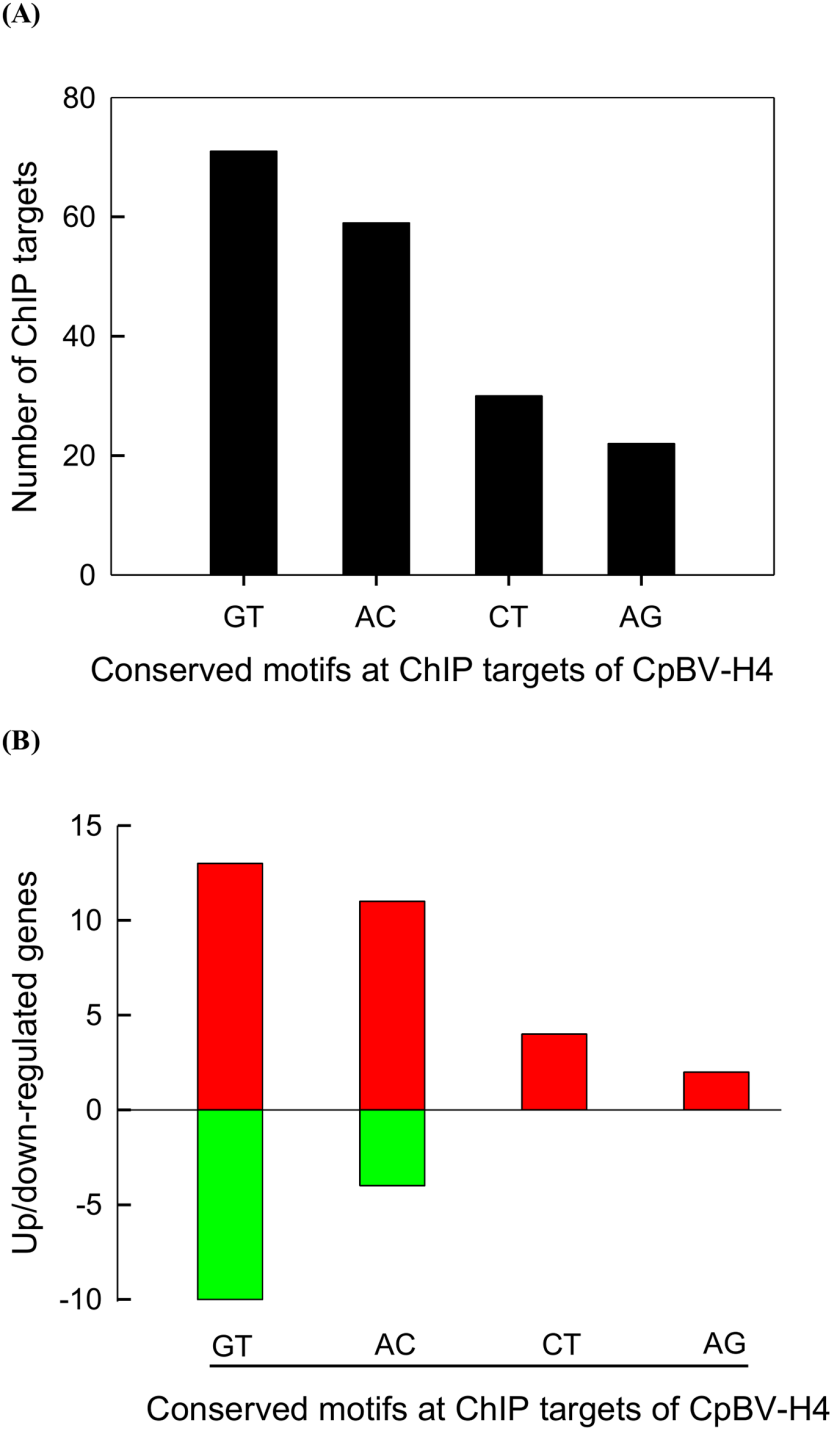
DNA sequence characters of CpBV-H4 joining sites on *P*. *xylostella* genome. (A) Occurrence of two nucleotide repeat motifs among ChIP targets (480 sites assessed) against chromatins extracted from host larvae parasitized by *C*. *plutellae*. (B) Expression profiles of genes close to CpBV-H4 joining sites containing repeat motifs. Up/down-regulated genes are defined by at least two-fold change in FPKM values.

Sequences of P7-specific ChIP targets (538 sites) were further analyzed in order to find any consensus sequences (Fig 4). For this analysis, 480 target sequences were used while the other 58 target sequences did not have unique match with *P*. *xylostella* genome. These 480 targets were rich (60.2%) in AT content. Almost 40% targets contained two nucleotide repeat sequences such as GT- (14.9%), AC- (12.4%), CT- (6.3%), and AG- (4.6%) repeats (Fig 4A and S2 Fig). Three nucleotide repeat sequences (CAT and TGA), four nucleotide repeat sequences (TACA, TCAC, TGAG, TCTG, GTCT, and TAGA), and five nucleotide repeat sequences (TTCTG, CAATA, and ATTCT) were also detected (S2 Fig). GT and AC repeat-joining sites were close to genes that were up- or down- regulated in their expression during *C*. *plutellae* parasitism (Fig 4B). In contrast, CT- and AG- repeat joining sites were associated with up-regulated genes. These repeats were located in 45.8% sites at the upstream (‘UP’), 20.8% sites at the downstream (‘DOWN’), and 33.3% sites at the gene body (‘GB’) (Table 4). Among these repeats, more than 50% CT- and AG- repeats were located at GB. Most repeat motifs were likely to be associated with DNA elements recognized by transcriptional factors or chromatin remodeling factors (S2 Table). Among the 51 core targets of CpBV-H4, more than 60% possessed GT- or AC- repeats.

**Table 4:** Characters of DNA sequences in 480 ChIP targets of CpBV-H4 against chromatins derived from *P*. *xylostella* parasitized by *C*. *plutellae*. ChIPs are localized at upstream (UP), downstream (DOWN), or gene body (GB) with respect to the nearest coding DNA sequence (CDS).

ChIP targets	Location to CDS	ChIP targets	Location to CDS	ChIP targets	Location to CDS
GT-repeat					
SC_01 (1529579–1529733)	UP	SC_139 (305106–305260)	DOWN	SC_75 (666947–667101)	DOWN
SC_04 (643998–644152)	UP	SC_141 (88466–88620)	UP	SC_83 (244226–244380)	DOWN
SC_07 (317692–317846)	UP	SC_143 (403725–403879)	UP	SC_90 (137739–137893)	UP
SC_11 (430487–430641)	DOWN	SC_143 (330068–330222)	UP	SC_98 (152754–152908)	DOWN
SC_12 (1683234–1683388)	GB	SC_147 (914126–914280)	GB	SC_99 (556050–556204)	UP
SC_18 (443738–443892)	GB	SC_150 (256771–256925)	UP	SC_104 (240347–240501)	DOWN
SC_19 (1411864–1412018)	GB	SC_159 (481456–481610)	GB	SC_106 (43556–43710)	UP
SC_19 (559394–559548)	GB	SC_170 (316051–316163)	UP	SC_482 (181374–181528)	UP
SC_20 (273829–273983)	GB	SC_177 (414187–414299)	GB	SC_505 (90668–90822)	UP
SC_20 (846120–846274)	GB	SC_196 (47489–47643)	DOWN	SC_523 (48915–49069)	UP
SC_23 (890357–890511)	UP	SC_202 (288112–288266)	UP	SC_628 (73207–73361)	GB
SC_28 (77042–77196)	UP	SC_227 (95085–95239)	UP	SC_666 (69847–70001)	UP
SC_28 (1292146–1292300)	DOWN	SC_236 (466266–466378)	UP	SC_730 (8408–8562)	DOWN
SC_29 (1141735–1141889)	DOWN	SC_245 (408514–408668)	DOWN	SC_730 (7933–8087)	UP
SC_32 (1125668–1125822)	DOWN	SC_275 (311126–311280)	UP	SC_108 (230141–230295)	DOWN
SC_33 (1120937–1121091)	DOWN	SC_287 (241760–241914)	GB	SC_120 (600098–600252)	DOWN
SC_35 (1079097–1079251)	UP	SC_294 (221145–221299)	DOWN	SC_120 (764303–764457)	UP
SC_35 (1210103–1210257)	GB	SC_294 (277399–277553)	UP	SC_125 (351176–351330)	GB
SC_43 (142314–142468)	UP	SC_330 (206688–206842)	UP	SC_762 (17422–17576)	UP
SC_45 (1750992–1751146)	UP	SC_330 (117417–117571)	GB	SC_809 (35039–35193)	DOWN
SC_48 (1140761–1140915)	DOWN	SC_351 (197374–197528)	UP	SC_817 (9094–9248)	UP
SC_52 (1467136–1467290)	UP	SC_373 (101515–101669)	UP	SC_849 (42367–42521)	UP
SC_59 (276702–276814)	DOWN	SC_404 (217526–217680)	DOWN	SC_460 (21458–21612)	DOWN
SC_74 (1093281–1093435)	UP				
AC-repeat					
SC_1 (1307832–1307986)	GB	SC_58 (525114–525268)	UP	SC_128 (250593–250747)	UP
SC_4 (838927–839081)	UP	SC_58 (525114–525268)	UP	SC_130 (736354–736508)	GB
SC_10 (388099–388253)	GB	SC_65 (302353–302507)	DOWN	SC_137 (178767–178921)	DOWN
SC_19 (1466517–1466671)	UP	SC_73 (1283224–1283378)	GB	SC_140 (271680–271834)	UP
SC_23 (994708–994862)	UP	SC_78 (288396–288550)	GB	SC_141 (239742–239896)	UP
SC_24 (708520–708632)	GB	SC_85 (679828–679982)	UP	SC_151 (107858–108012)	GB
SC_30 (1553838–1553992)	DOWN	SC_93 (249512–249666)	UP	SC_138 (756685–756839)	GB
SC_34 (49087–49241)	UP	SC_101 (385648–385802)	UP	SC_140 (884673–884827)	UP
SC_36 (91667–91821)	DOWN	SC_107 (4297–4451)	UP	SC_169 (507650–507804)	DOWN
SC_37 (245401–245513)	UP	SC_111 (321919–322073)	UP	SC_287 (64942–65096)	GB
SC_42 (822155–822309)	GB	SC_117 (237010–237164)	UP	SC_215 (123683–123837)	UP
SC_48 (385357–385511)	GB	SC_123 (228409–228563)	GB	SC_224 (280319–280473)	GB
SC_225 (89742–89896)	UP	SC_351 (266539–266693)	UP	SC_455 (312221–312375)	DOWN
SC_251 (383566–383720)	GB	SC_356 (108626–108780)	UP	SC_478 (83062–83216)	GB
SC_296 (330810–330964)	UP	SC_362 (262500–262654)	UP	SC_543 (110836–110948)	UP
SC_314 (22999–23153)	DOWN	SC_388 (61617–61771)	GB	SC_699 (70174–70328)	UP
SC_315 (65035–65189)	UP	SC_428 (145021–145175)	UP	SC_737 (46820–46974)	DOWN
SC_318 (179793–179947)	GB	SC_437 (153817–153971)	DOWN	SC_760 (17952–18106)	GB
SC_336 (207567–207721)	DOWN	SC_447 (284887–285041)	UP	SC_763 (28897–29051)	GB
SC_1204 (9889–10043)	UP	SC_1312 (9571–9654)	UP		
CT-repeat					
SC_1 (1526810–1526964)	GB	SC_56 (928618–928772)	GB	SC_299 (252501–252655)	GB
SC_22 (1342063–1342217)	DOWN	SC_78 (54226–54380)	GB	SC_305 (364488–364642)	UP
SC_36 (583812–583966)	GB	SC_143 (33368–33522)	GB	SC_322 (338167–338321)	DOWN
SC_36 (546705–546859)	GB	SC_160 (367598–367710)	UP	SC_322 (337312–337466)	UP
SC_36 (1076285–1076439)	UP	SC_172 (353055–353209)	DOWN	SC_349 (108599–108753)	GB
SC_43 (724320–724474)	GB	SC_173 (146979–147133)	GB	SC_743 (30023–30177)	GB
SC_48 (149295–149449)	UP	SC_177 (429466–429620)	UP	SC_809 (35039–35193)	UP
SC_54 (720397–720551)	GB	SC_241 (136600–136754)	DOWN	SC_878 (9939–10093)	DOWN
SC_1204 (9889–10043)	DOWN	SC_254 (343736–343890)	UP	SC_1179 (14521–14675)	GB
SC_143 (33368–33522)	UP	SC_293 (594457–594569)	GB	SC_1204 (7646–7801)	GB
AG-repeat					
SC_9 (215394–215548)	GB	SC_116 (483326–483480)	UP	SC_177 (386088–386242)	GB
SC_42 (155599–155753)	UP	SC_140 (877781–877935)	UP	SC_440 (53487–53641)	GB
SC_43 (63654–63808)	GB	SC_168 (307280–307434)	GB	SC_98 (30140–30294)	DOWN
SC_48 (63311–63465)	DOWN	SC_175 (1067104–1067258)	UP	SC_133 (18157–18311)	GB
SC_83 (591625–591779)	UP	SC_496 (158094–158248)	GB	SC_97 (310848–311002)	GB
SC_87 (532810–532964)	GB	SC_762 (17422–17576)	GB	SC_478 (60041–60195)	GB
SC_97 (311398–311552)	UP	SC_1291 (5757–5911)	UP		
Three to five nucleotide repeats					
SC_71 (1089368–1089522)	UP	CAT-repeat			
SC_108 (843558–843712)	UP	TGA-repeat			
SC_32 (499690–499844)	GB	TACA-repeat			
SC_12 (632499–632653)	UP	TGAG-repeat			
SC_1 (1547821–1547975)	GB	TACA-repeat			
SC_160 (542173–542327)	UP	TCAC-repeat			
SC_852 (38866–39020)	UP	GTCT-repeat			
SC_444 (59391–59545)	UP	TCTG-repeat			
SC_67 (536043–536197)	UP	TCTG-repeat			
SC_223 (357500–357654)	DOWN	TAGA-repeat			
SC_36 (583812–583966)	GB	ATTCT-repeat			
SC_36 (583812–583966)	GB	TTCTG-repeat			
SC_592 (112905–113059)	GB	CAATA-repeat			

### Physical mapping of 51 core joining sites of CpBV-H4 on host chromosomes

**Fig 5:**
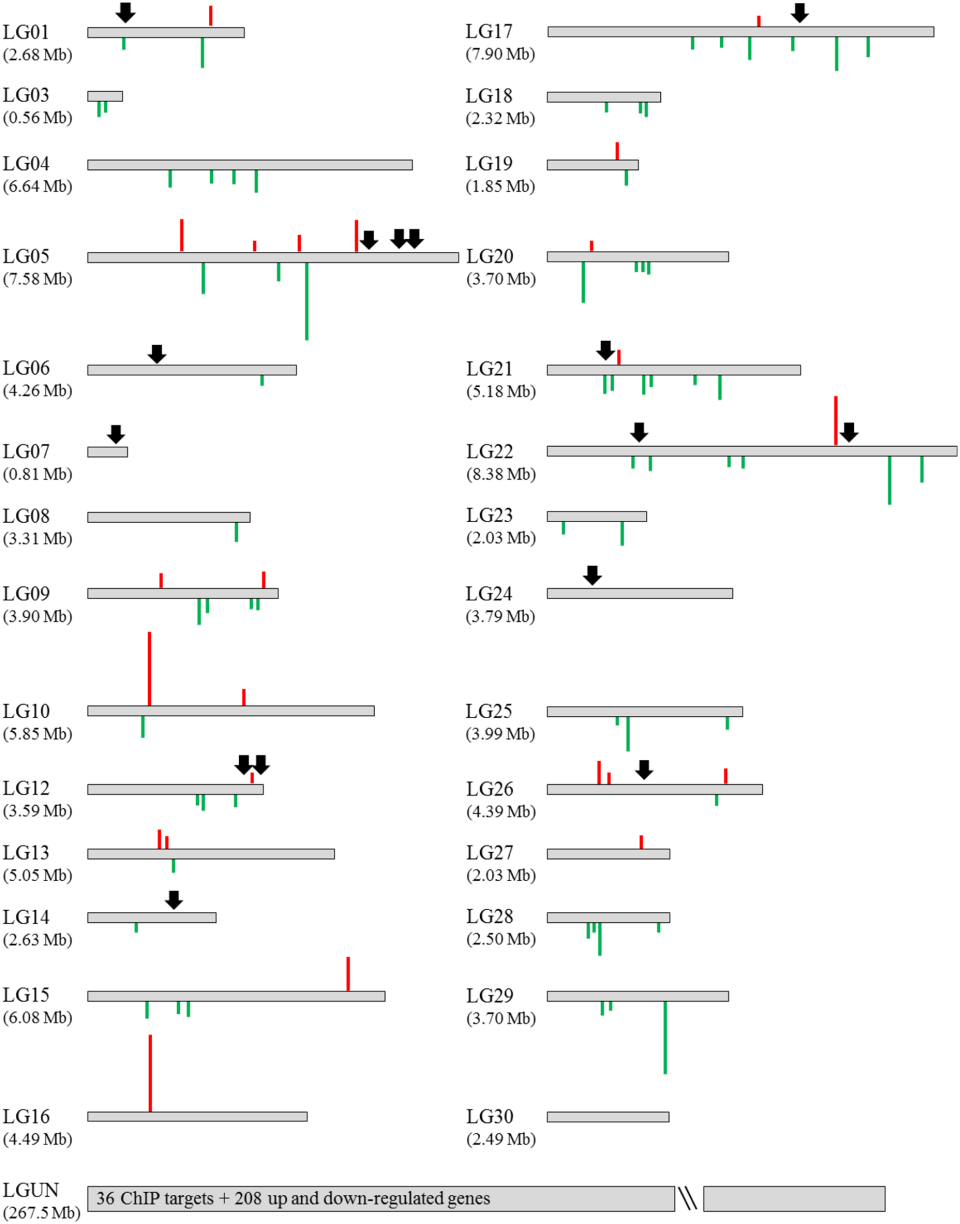
Physical mapping of 51 CpBV-H4 joining sites on *P*. *xylostella* chromosomes. A total of 29 chromosomes are presented in linkage groups (‘LGs’). Of the 51 CpBV-H4 joining sites, 15 sites (down arrows) are denoted on 11 LGs while the other 36 joining sites are localized in uncharacterized LG (‘LGUN’). Differentially expressed genes (DEGs) after by *CpBV-H4* expression are denoted by red (up-regulated genes) and green (down-regulated genes) bars on LGs. DEGs are defined by more than 2-fold change in FPKM values.

The 51 core joining sites of CpBV-H4 were mapped onto the chromosomes of *P*. *xylostella* (Fig 5). *P*. *xylostella* chromosomes have been characterized with 30 linkage groups and uncharacterized scaffolds [18]. Fifteen CpBV-H4 joining sites were located on 11 chromosomes while the remaining 36 CpBV-H4 sites were found on uncharacterized chromosome(s). On this mapping, 302 host DEGs regulated by CpBV-H4 during *C*. *plutellae* parasitism were applied (see vertical lines in Fig 5). These target genes were not only distributed on chromosomes containing CpBV-H4 targets, but also distributed on other chromosomes without CpBV-H4 targets.

### Influence of relative locality of CpBV-H4 targets on expression of nearby genes

**Fig 6:**
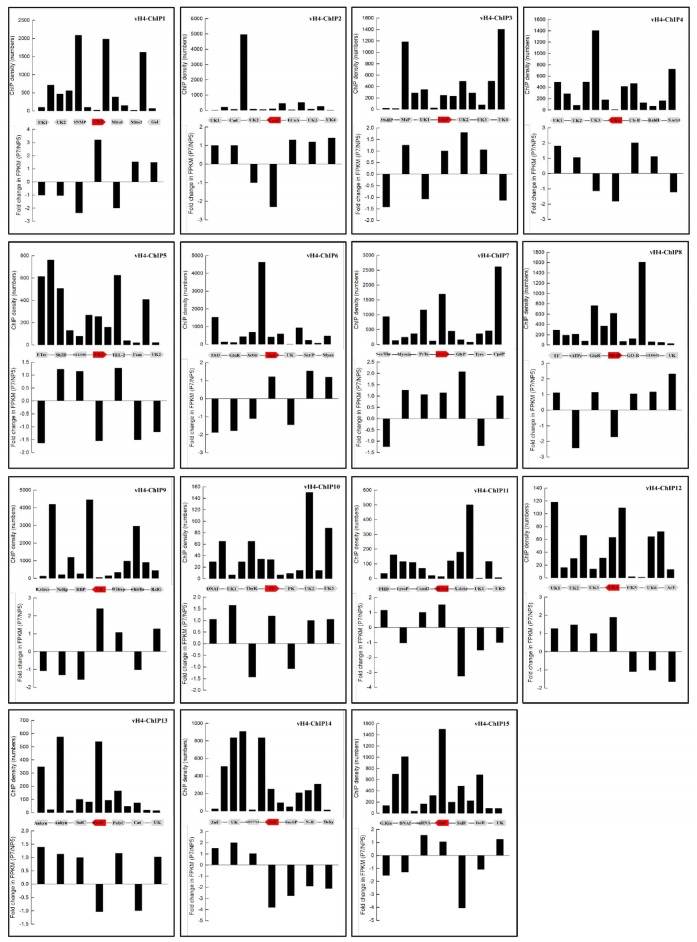
Distribution of CpBV-H4 ChIPs around the 15 joining sites of CpBV-H4 on *P*. *xylostella* genome. In each joining site, six neighboring genes around a main target site denoted by red-colored gene were selected. Absolute numbers of ChIP reads were counted on six gene bodies and their intergenic regions. Changes in gene expression levels (FPKM values) of these seven genes after *CpBV-H4* expression were depicted in lower graph of each panel, in which negative sign indicates decreased gene expression. All gene names and acronym are described in S4 Table.

**Fig 7:**
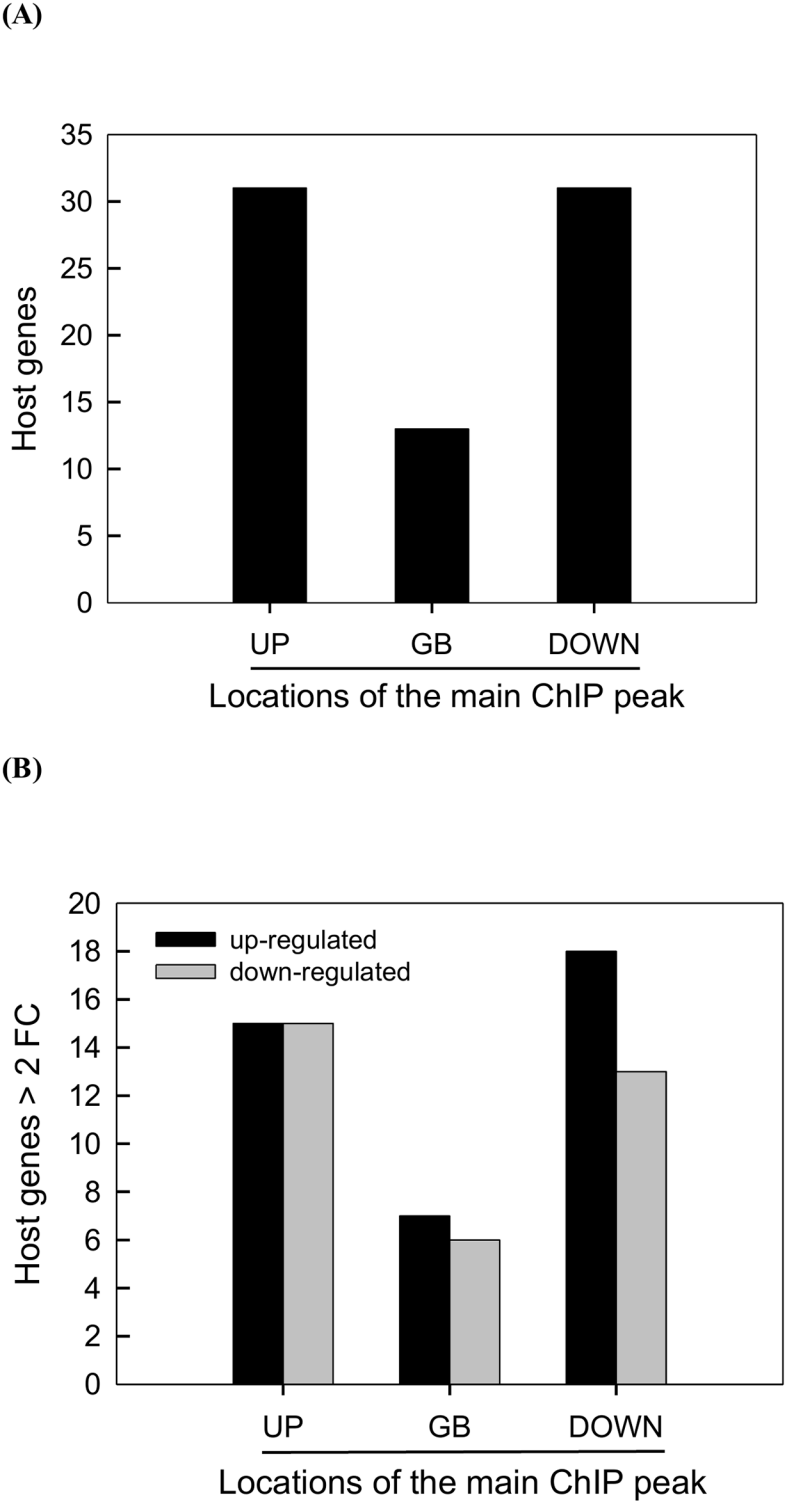
Positional effect of the 15 CpBV-H4 joining sites on regulation of host gene expression. ChIPs against CpBV-H4 were counted on gene body (‘GB’), upstream (‘UP’), and downstream (‘DOWN’). (A) Frequency of CpBV-H4 ChIPs around 75 target genes. (B) Number of host genes differentially regulated in expression level after CpBV-H4 expression in their three different genic regions. Differentially expressed genes are defined by at least 2-fold change (FC) in FPKM levels. Gene names and acronym are listed in S4 Table.

We then tested whether there was any positional effect of CpBV-H4 targets on regulating gene expression of nearby genes. To address this question, 15 CpBV-H4 targets (‘vH4-ChIP1 ~ vH4-ChIP15’) were further divided into three relative localities in each nearby gene on coding DNA sequence or gene body (‘GB’), upstream intergenic region (‘UP’), and downstream intergenic region (‘DOWN’). Subsequently, when relative localities and expressional changes of nearby genes induced by *CpBV-H4* expression were compared, individual ChIPs were not evenly distributed on all tested genes. They were concentrated around target gene sites (red color in (Fig 6) except vH4-ChIP2 and vH4-ChIP3. This might be due to the fact that target genes were determined by ChIP frequency in a specific spot, not by total ChIP frequency in each locality. Host genes around CpBV-H4 targets exhibited expressional changes (either up- or down- regulated). Relative distribution of CpBV-H4 target sites was further analyzed to determine any positional effect on regulating the expression level of nearby genes (Fig 7). In this analysis, 75 neighboring genes including 15 CpBV-H4-joining sites were assessed. In these CpBV-H4 joining sites, ChIP targets were relatively evenly distributed on three positions (Fig 7A). The less number of major ChIPs by about two folds of change at GB might be due to difference in domain size because the average GB domain length (≈ 1.5 kb) was shorter by almost two folds than UP (≈ 2.5 kb) and DOWN (≈ 3.0 kb) domain lengths. Each gene was classified by the presence of the main ChIP peak among the three relative localities (S3 Table and Fig 7B). Although different genes had different locations of main ChIP peaks, the three ChIP positions did not have significant effect on the direction of gene regulation (X^2^ = 0.3995; df = 2; *P* = 0.8189).

## Discussion

Parasitism by endoparasitoid wasps using PDV alters host gene expression to allow internal environment to be favorable for parasite development [19]. Two different endoparasitoid wasps, *Diadegma semiclausum* and *C*. *plutellae* possessing IV and BV, respectively, can parasitize *P*. *xylostella* and significantly alter host gene expression [20, 21]. Several viral factors have been proposed to be able to regulate host gene expression in CpBV [10]. CpBV-IkB is a viral IkB possessing ankyrin repeat. However, it lacks regulatory domain and suppresses antimicrobial peptide gene expression in response to immune challenge [22]. Two homologous proteins, CpBV15α and CpBV15β, can interrupt eukaryotic translation initiation factors and act as translational inhibitory factors against mRNAs of *P*. *xylostella* [16, 23]. Recently, a cys-motif protein of CpBV has been reported to be able to suppress the translation of host mRNAs [24]. CpBV-H4 is known to be able to regulate host gene expression in transcriptional level by an epigenetic mode [14, 16]. Thus, parasitism of *C*. *plutellae* can regulate host gene expression at both transcription level and translational level.

This study determined how many host genes of *P*. *xylostella* were regulated at transcriptional level by *C*. *plutellae* parasitism using RNA-Seq. In addition, this study determined the contribution of CpBV-H4 to host genes regulated by *C*. *plutellae* parasitism. DEG analysis of transcriptomes between parasitized and nonparasitized larvae at late stage determined host genes regulated by parasitism, in which 877 genes were up-regulated and 981 genes were down-regulated. These regulated host genes were predicted to have various functional categories including metabolism, development, immunity, signaling, and gene expression regulator. Venom proteins, ovarian proteins, teratocyte, and CpBV are known to be parasitic factors of *C*. *plutellae* [25, 26]. Venom and ovarian proteins produced from female wasp are delivered to parasitized host along with eggs to suppress host immunity or help PDV enter target cells [27, 28]. It has been suggested that these two factors play crucial roles during early parasitic stage [29]. However, the current study analyzed host gene expression at late parasitic stage. Thus, the altered host gene expression might be induced mainly by teratocyte and CpBV. It has been reported that *P*. *xylostella* larvae injected with teratocytes exhibit significant difference in expression pattern compared to untreated control larvae [26]. *P*. *xylostella* larvae injected with CpBV also can suppress host gene expression [30]. Especially, an SSH analysis has shown that CpBV-H4 alone can inhibit the expression levels of at least 115 host genes at transcription level [14]. Thus, the 1,858 host genes regulated by *C*. *plutellae* parasitism found in this study might have been influenced by both teratocyte and CpBV. Furthermore, this current study was focused on the single influence of CpBV-H4 on host gene regulation and how much it contributed to parasitism with respect to host gene regulation. Using the *in vivo* transient expression technique used to determine physiological functions of PDV genes in a previous study [31], CpBV-H4 was transiently expressed in nonparasitized larvae in this study. A truncated CpBV-H4 prepared by removing 38 amino acid residues at the N-terminal tail used in a previous study [11] was also expressed in the same aged larvae as a control. DEG analysis of these two transient expression groups resulted in 1,190 host genes specifically regulated in their expressions by CpBV-H4. Finally, the comparison of 1,858 genes regulated by parasitism and 1,190 genes regulated by CpBV-H4 showed that 302 host genes were overlapped in the two groups. Thus, 16.8% of host genes regulated by *C*. *plutellae* parasitism at late parasitic stage were induced by CpBV-H4 expression while the other 83.2% genes might have been regulated by other CpBV factors and teratocyte. Our current analysis supported the results of previous SSH analysis [14] because most (95%) down-regulated genes (115 genes) determined by SSH analysis were included in the down-regulated genes found in the current RNA-Seq analysis. In contrast, the remaining 888 host genes influenced by CpBV-H4 expression but not included in genes regulated by *C*. *plutellae* parasitism might be induced by unnatural effect of CpBV-H4 on host regulation such as excess amount (25 ng CpBV-H4 per larva) of gene dose compared to natural parasitism (< 1 ng per larva [32] or the absence of interacting effect with other parasitic factors.

CpBV-H4 was mainly localized in 51 joining sites based on ChIP-Seq analysis. These joining sites were determined by overlapping sites based on the nearest genes between joining sites determined from parasitized larvae and larvae transiently expressing CpBV-H4. In-depth analysis of these joining sites showed that individual ChIP sites were unevenly distributed around target genes. However, this biased distribution did not influence the direction (up or down) of gene regulation. Thus, target genes can be up- or down- regulated irrespective to biased distribution of CpBV-H4 on upstream, gene body, or downstream locations. A previous study [17] pointed out that CpBV-H4s are localized on promoter regions of inducible target genes which might be highly involved in chromatin remodeling. However, the current ChIP-Seq analysis showed that CpBV-H4 could be integrated into gene body or downstream as well as upstream possessing promoter regions.

Physical mapping of joining sites of CpBV-H4 showed that CpBV-H4s were localized mainly into a small number of chromosomes. In contrast, host genes regulated by CpBV-H4 were not close to CpBV-H4 joining sites. Some target genes controlled by CpBV-H4 were localized on chromosomes in which CpBV-H4 was not integrated. This suggests that genes regulated by CpBV-H4 might be able to regulate subsequent target genes by acting as transcriptional regulators. Indeed, target genes regulated by CpBV-H4 included transcription regulators involved in gene expression. For example, the expression levels of Px-KDM and Px-SWI/SNF were suppressed by CpBV-H4 in both SSH and RNA-Seq analyses. KDM and SWI/SNF are known to be able to regulate other gene transcription in an epigenetic mode [33, 34]. Furthermore, these two genes are required for immunity and development of *P*. *xylostella* [14]. Thus, CpBV-H4 can influence host gene expression by directly binding to chromatin around target genes or by indirectly influencing host gene expression by regulating transcriptional regulators.

This study found that 302 host genes were regulated by CpBV-H4 with joining sites near 51 target genes. Furthermore, this study explained a significant contribution (16.8%) of CpBV-H4 on host gene regulation induced by *C*. *plutellae* parasitism because teratocytes and other 156 CpBV genes might play crucial roles in altering host gene expression. Similar viral histone H4s have been found in other *Cotesia*-associated BVs [35]. Thus, viral histone H4 might be a main parasitic factor in *Cotesia*-associated parasitism.

## Materials and methods

### Insects and parasitization

*P*. *xylostella* larvae were reared with cabbage leaves at 25 ± 1°C with photoperiod of 16:8 h (L:D). Adults were fed 10% sucrose. Late first instar larvae were parasitized by *C*. *plutellae* at 1:2 (wasp: host) density for 24 h under the rearing condition. Parasitized larvae were fed cabbage leaves. Parasitized larvae lived for 8 days (P1–P8). They died after the emergence of fully matured wasp larvae which shortly formed cocoons for pupal development. In contrast, Nonparasitized larvae at the age corresponding to P larvae at parasitization lived 6 days (NP1–NP6) at 25°C and pupated. Thus, P1 was an equivalent age to NP1 while P7 was an equivalent age to P7. Wasp cocoons were collected and kept at 25°C for adult tissue development. After emergence, adult wasps were allowed to mate for 24 h. They were then used for parasitization.

### Transient expression of CpBV-H4 in *P*. *xylostella* larvae

A gene encoding CpBV-H4 containing N-terminal tail (vH4) and truncated CpBV-H4 (vH4T) after deleting N-terminal tail was cloned into a pIB vector [17]. The recombinant plasmid (250 ng/μL) was mixed in an equal volume of Metafectene PRO transfection reagent (Biontex, Planegg, Germany) and incubated for 20 min at room temperature to allow the formation of DNA-lipid complex. A total of 100 nL of this DNA-lipid complex was injected into the hemocoel of NP3 larvae at a rate 10 nL/sec using microsyringe pump controller (PV830 Pneumatic Pico Pump, World Precision Instruments, Sarasota, FL, USA) under a microscope (Olympus S730, Tokyo, Japan). At 48 h post-infection, transient expression of vH4 was confirmed by RT-PCR using forward primer 5′–GGATCCATGGCTGATCATCCTAAAGG–3′ and reverse primer 5′–GAATTCTCAACCTCCATAACCATAGATC–3′. The expression of vH4T was determined using forward primer 5′–GGATCCATGGGAAGAGGATTGGGCAA–3′ and reverse primer 5′–GAATTCTCAACCTCCATAACCATA GATC–3′. Total RNA of larvae expressing vH4 or vH4T was extracted using Trizol reagent described below for subsequent RNA-Seq analysis.

### RNA extraction for RNA-Seq analysis

Total RNA was isolated from four groups of *P*. *xylostella* larvae (P7, NP5, vH4-expressing larvae, and vH4T-expressing larvae) using Trizol reagent (Invitrogen, Carlsbad, CA, USA). Extracted RNA was resuspended in 40 μL of diethyl pyrocarbonate-treated water. RNA integrity for subsequent RNA-Seq was analyzed using Bioanalyzer 2100 (Agilent Technologies, Santa Clara, CA, USA). The RNA QC was evaluated based on RNA Integrity Number (RIN) value greater than or equal to 7. Our samples had RIN values of 7.7 to 8.5.

Using these total RNAs, cDNA library was constructed with Truseq RNA kit (Invitrogen, Seoul, Korea) and sequenced on Illumina HiSeq 2000 platform (Illumina Inc., San Diego, CA, USA) with 101 bp pair-end sequencing. Raw reads were trimmed using Trimmomatic 0.32 program (http://www.usadellab.org/cms/?page=trimmomatic) under a criterion of 230 (phred score base quality 30% or more). They were then mapped onto DBM genome (http://iae.fafu.edu.cn/DBM/index.php) using TopHat program (version 2.0.13) (http://ccb.jhu.edu/software/tophat/index.shtml) and Bowtie (version 2 2.2.3). From these mappings, NP5, P7, transient expression of CpBV-H4, and transient expression of truncated CpBV-H4 samples had 55.2%, 26%, 51.9%, and 52.1% mapping ratios, respectively (S1 Table). These mapped reads were then assembled with Cufflinks (version 2.2.1) (http://cole-trapnell-lab.github.io/cufflinks/). The assembled contigs were used to calculate FPKM (fragment per kilobase of transcript per million mapped reads). They were then annotated with DBM database.

### Differentially expressed gene (DEG) transcript analysis

For DEG analysis of four treatment groups described in section 2.3, a total of 12,945 transcripts were used by deleting 5,128 transcripts from a total of 18,073 transcripts because at least one of these samples contained FPKM values of 0. DEG used a criterion of at least two-fold change in FPKM value.

### Chromatin immunoprecipitation and deep sequencing (ChIP-Seq)

ChIP-Seq was performed using the above-mentioned four treatment groups of *P*. *xylostella*. A polyclonal antibody [17] for CpBV-H4 was used for ChIP. Briefly, 50 larvae for each group were homogenized in 1% sodium dodecyl sulphate followed by addition of formaldehyde (final concentration of 1%) to fix DNA with protein and then sonicated for 30 cycles for 1 min with 2 min interval. After sonication, DNA fragments in the range of 200–300 bp in 200 μL were used for ChIP-Seq according to QuickChIP kit manual (Cat. No. NBP2-29902, Novus Biologicals, Littleton, CO, USA). Briefly, 200 μL of sheared DNA was added to 800 μL of ChIP dilution buffer containing 75 μL of salmon sperm DNA (Sigma, St. Louis, MO, USA). CpBV-H4 antibody (1 μg) was added along with Protein A/G agarose (Thermo Scientific, Waltham, MA, USA). The complex of captured chromatin was then dissociated into protein and DNA using Tris-EDTA buffer (10 mM Tris, 1 mM EDTA, pH 8.0). The precipitated DNA was then resuspended in the same Tris-EDTA buffer and subjected to sequencing using Illumina HiSeq2000 platform with paired-end sequencing mode.

### Screening of major incorporation sites of CpBV-H4 on host chromatin from ChIP-Seq

Four ChIP-Seq data were used to determine CpBV-H4 joining sites. All ChIP sites were validated by significance value to discriminate specific and nonspecific bindings. To compare P7 and NP5 ChIP-Seq sites, significance at E value of 10^−8^ was used. Remaining validated ChIP sites were annotated by their nearest genes. Parasitism-specific ChIP sites were determined by manually deleting overlapping genes between two ChIP sites derived from P7 and NP5 larvae. To compare vH4 and vH4T ChIP-Seq sites, significance at E value of 10^−13^ was used due to relatively higher mapping ratios compared to that in the comparison between P7 and NP5 ChIPs. Remaining validated ChIP sites were annotated by their nearest genes. CpBV-H4-specific ChIP sites were determined by manually deleting overlapping genes between vH4 and vH4T ChIP sites. Major incorporation sites of CpBV-H4 were then determined by overlapping sites between parasitism-specific ChIP sites and CpBV-H4-speicific ChIP sites.

### Physical mapping of ChIP targets on *P*. *xylostella* chromosome

Total chromosomal map (394 Mb) assigned into 31 linkage groups (LGs) was downloaded from DBM database (http://ise.fafu.edu.DBM/chrList.php) divided into 171 scaffolds (111.9 Mb). For physical mapping, locations of all 51 ChIP targets were identified using FGENESH program (http://www.softberry.com). The ORFs of *P*. *xylostella* were predicted using *Bombyx mori* ORFs as templates. All 51 CpBV-H4 ChIP-Seq targets were physically mapped onto 31 LGs of *P*. *xylostella*.

### In-depth localization analysis of major incorporation sites of CpBV-H4

Among 51 ChIP-Seq targets with respect to CpBV-H4, 15 targets were located on 11 characterized LGs. In-depth analyses of these 15 CpBV-H4 ChIP-Seq targets were done by choosing three ORF nearest to both sides of main ChIP-Seq targets. The total numbers of ChIPs were then counted on gene body (‘GB’, a region containing open reading frame), 5’ region (‘upstream’) from GB, and 3’ region (‘downstream’) from GB, in which downstream and upstream regions were intergenic regions. The fold changes in FPKM for all 105 ORFs were obtained from RNA-Seq DEG data between P7 vs NP5.

### Screening of parasitism-specific and CpBV-H4-specific genes

Four treatment group samples were used for the selection of parasitism-specific (P7 vs NP5) and CpBV-H4-specific (vH4 vs vH4T) genes. The total number of genes expressed in both groups was separated based on up-regulation or down-regulation. In each regulation group, genes exhibiting more than two-fold change were chosen. These genes were then compared to the overlapped genes between P7 and NP5 to select parasitism-specific genes or compared to overlapped genes between vH4 and vH4T to select CpBV-H4-specific genes.

### ChIP sequence analysis for CpBV-H4 targets against P7 chromatins

Joining sites (133–155 nucleotides) of CpBV-H4 against chromatins of parasitized larvae were further selected based on E-value criterion at 10^−8^. All 538 target sequences were then aligned using MegAlign of Lasergene to identify repeat sequences and consensus motifs. AT ratios of targets were estimated using ENDMEMO program (http://www.endmemo.com/bio/gc.php). Consensus sequences were functionally annotated from DBM database (http://iae.fafu.edu.cn/DBM/index.php) using MOTIF program (www.genome.jp/tools/motif/).

## Supporting information

S1 Table RNA-Seq summary of different treatment groups of *P*. *xylostella* larvae. Treatments includes *in vivo* transient expression of CpBV-H4 (‘vH4’) or truncated CpBV-H4 (‘vH4T’), parasitized larvae at day 7 (‘P7’) or nonparasitized larvae at day 5 (‘NP5’).
(DOCX)

S2 Table Functional prediction of repeat motifs found in CpBV-H4 joining sites. The prediction used MOTIF program (www.genome.jp/tools/motifs/).
(DOCX)

S3 Table Influence of distribution of *CpBV-H4* joining sites on expression of nearest genes. The *CpBV-H4* location was determined by the most significant ChIP assay. The locations of gene body (‘GB’), UPSTREAM (‘UP’), and downstream (‘DOWN’) were with respect to the nearest coding DNA sequence (CDS).
(DOCX)

S4 Table Full name and their acronyms used in Fig 6. (DOCX)

S1 Fig Number of ChIP-Seq targets in four different treatments including *in vivo* transient expression of CpBV-H4 (‘vH4’), truncated CpBV-H4 (‘vH4T’), parasitized larvae at day 7 (‘P7’), and nonparasitized larvae at day 5 (‘NP5’). (TIF)

S2 Fig Repeat sequences in some joining sites of CpBV-H4. (TIF)

## References

[1] Webb BA, Beckage NE, Hayakawa Y, Krell PJ, Lanzrein B, Stoltz DB, et al (2000) Polydnaviridae In Virus taxonomy (van Regenmortel MHV, Maniloff J, Mayo MA, McGeoch DJ, Preingle CR & Wickner RB, eds.), pp. 253–260. Academic Press, New York.

[2] Bezier A, Herbiniere J, Lanzrein B, Drezen JM (2009) Polydnavirus hidden face: the genes producing virus particles of parasitic wasps. J Invertebr Pathol 101: 194–203. 10.1016/j.jip.2009.04.006 19460382

[3] Strand MR, Burke GR (2013) Polydnavirus-wasp associations: evolution, genome organization, and function. Curr Opin Virol 3: 587–594. 10.1016/j.coviro.2013.06.004 23816391

[4] Burke GR, Strand MR (2014) Systematic analysis of a wasp parasitism arsenal. Mol Ecol 23: 890–901. 10.1111/mec.12648 24383716 PMC4120856

[5] Bae S, Kim Y (2004) Host physiological changes due to parasitism of a braconid wasp, *Cotesia plutellae*, on diamondback moth, *Plutella xylostella*. Comp Biochem Physiol A 138: 39–44. 10.1016/j.cbpb.2004.02.018 15165569

[6] Ibrahim AM, Kim Y (2006) Parasitism by *Cotesia plutellae* alters the hemocyte population and immunological function of the diamondback moth, *Plutella xylostella*. J Insect Physiol 52: 943–950. 10.1016/j.jinsphys.2006.06.001 16872627

[7] Kwon B, Song S, Choi JY, Je YH, Kim Y (2010) Transient expression of specific *Cotesia plutellae* bracoviral segments induces prolonged larval development of the diamondback moth, *Plutella xylostella*. J Insect Physiol 56: 650–658. 10.1016/j.jinsphys.2010.01.013 20138886

[8] Kumar S, Kim Y. Revision of Cotesia plutellae bracovirus genes and RNA-Seq: their persistent expression in infected host. Insect Sci (submitted).

[9] Burke GR, Strand MR (2015) Polydnaviruses: from discovery to current insights. Virology 10: 393–402. 10.1016/j.virol.2015.01.018 PMC4424053 25670535

[10] Kim Y, Choi Y, Je YH (2007) Cotesia plutellae bracovirus genome and its function in altering insect physiology. J Asia Pac Entomol 10: 181–191.

[11] Gad W, Kim Y (2008) A viral histone H4 encoded by Cotesia plutellae bracovirus inhibits haemocyte-spreading behaviour of the diamondback moth, *Plutella xylostella*. J Gen Virol 89: 931–938. 10.1099/vir.0.83585-0 18343834

[12] Gad W, Kim Y (2009) N-terminal tail of a viral histone H4 encoded in Cotesia plutellae bracovirus is essential to suppress gene expression of host histone H4. Insect Mol Biol 18: 111–118. 10.1111/j.1365-2583.2009.00860.x 19196351

[13] Hepat R, Kim Y (2011) Transient expression of a viral histone H4 inhibits expression of cellular and humoral immune-associated genes in *Tribolium castaneum*. Biochem Biophys Res Commun. 2011; 415: 279–283. 10.1016/j.bbrc.2011.10.040 22037579

[14] Kumar S, Venkata P, Kim Y. Suppressive activity of a viral histone H4 against two host chromatin remodeling factors: lysine demethylase and SWI/SNF. J Gen Virol. 2016; 97: 2780–2796. 10.1099/jgv.0.000560 27443988

[15] Kim G, Kim Y. Up-regulation of circulating hemocyte population in response to bacterial challenge is mediated by octopamine and 5-hydroxytryptamine via Rac1 signal in *Spodoptera exigua*. J Insect Physiol. 2010; 56: 559–566. 10.1016/j.jinsphys.2009.11.022 19961854

[16] Hepat R, Kim Y. A viral factor, CpBV15α, interacts with a translation initiation factor, eIF2, to suppress host gene expression at a post-transcriptional level. J Invertebr Pathol. 2013; 114: 34–41. 10.1016/j.jip.2013.05.004 23711415

[17] Hepat R, Song JJ, Lee D, Kim Y. A viral histone h4 joins to eukaryotic nucleosomes and alters host gene expression. J Virol. 2013; 87: 11223–11230. 10.1128/JVI.01759-13 23926351 PMC3807275

[18] Baxter SW, Davey JW, Johnston JS, Shelton AM, Heckel DG, Jiggins CD, et al Linkage Mapping and Comparative Genomics Using Next-Generation RAD Sequencing of a Non-Model Organism. PLoS ONE. 2011; 6: e19315 10.1371/journal.pone.0019315 21541297 PMC3082572

[19] Provost B, Jouan V, Hilliou F, Delobel P, Bernardo P, Ravallec M. et al Lepidopteran transcriptome analysis following infection by phylogenetically unrelated polydnaviruses highlights differential and common responses. Insect Biochem Mol Biol. 2011; 41: 582–591. 10.1016/j.ibmb.2011.03.010 21457783

[20] Etebari K, Palfreyman RW, Schlipalius D, Nielsen LK, Glatz RV, Asgari S. Deep sequencing-based transcriptome analysis of *Plutella xylostella* larvae parasitized by *Diadegma semiclausum*. BMC Genomics 12: 446 10.1186/1471-2164-12-446 21906285 PMC3184118

[21] Song KH, Jung MK, Eum JH, Hwang IC Han SS (2008) Proteomic analysis of parasitized *Plutella xylostella* larvae plasma. J Insect Physiol 54: 1270–1280. 10.1016/j.jinsphys.2008.06.010 18671979

[22] Bae S, Kim Y (2009) IkB genes encoded in Cotesia plutellae bracovirus suppress an antiviral response and enhance baculovirus pathogenicity against the diamondback moth, *Plutella xylostella*. J Invertebr Pathol 102: 79–87. 10.1016/j.jip.2009.06.007 19559708

[23] Surakasi VP, Nalini M, Kim Y (2011) Host translational control of a polydnavirus, Cotesia plutellae bracovirus, by sequestering host eIF4A to prevent formation of a translation initiation complex. Insect Mol Biol 20: 609–618. 10.1111/j.1365-2583.2011.01091.x 21699595

[24] Kim E, Kim Y (2016) Translational control of host gene expression by a cys-motif protein encoded in a bracovirus. PLoS ONE 11: e0161661 10.1371/journal.pone.0161661 27598941 PMC5012692

[25] Basio NAM, Kim Y (2006) Additive effect of teratocyte and calyx fluid from Cotesia plutellae on immunosuppression of *Plutella xylostella*. Physiol Entomol 31: 341–347.

[26] Ali R, Kim Y (2013) Teratocyte-secreting proteins of an endoparasitoid wasp, *Cotesia plutellae*, prevent host metamorphosis by altering endocrine signals. J Invertebr Pathol 166: 251–262. 10.1016/j.cbpa.2013.06.028 23830810

[27] Webb BA, Luckhart S (1996) Evidence for an early immunosuppressive role for related *Campoletis sonorensis* venom and ovarian proteins in *Heliothis virescens*. Arch Insect Biochem Physiol 26: 147–163. 10.1002/arch.940260208 8054661

[28] Asgari S (2006) Venom proteins from polydnavirus-producing endoparasitoids: their role in host-parasite interactions. Arch Insect Biochem Physiol 61: 146–156. 10.1002/arch.20109 16482579

[29] Luckhart S, Webb BA (1996) Interaction of a wasp ovarian protein and polydnavirus in host immune suppression. Dev Comp Immunol 20: 1–21. 8738933 10.1016/0145-305x(95)00040-z

[30] Shi M, Zhao S, Wang ZH, Stanley D, Chen XX (2016) *Cotesia vestalis* parasitization suppresses expression of a *Plutella xylostella* thioredoxin. Insect Mol Biol. 25: 679–688. 10.1111/imb.12252 27376399

[31] Hepat R, Kim Y (2012) *In vivo* transient expression for the functional analysis of polydnaviral genes. J Invertebr Pathol 111: 152–159. 10.1016/j.jip.2012.07.025 22884446

[32] Kim J, Hepat R, Lee D, Kim Y (2013) Protein tyrosine phosphatase encoded in Cotesia plutellae bracovirus suppresses a larva-to-pupa metamorphosis of the diamondback moth, *Plutella xylostella*. Com Biochem Physiol A 166: 60–69. 10.1016/j.cbpa.2013.04.025 23651929

[33] Black JC, Allen A, Van RC, Forbes E, Longworth M, Tschöp K. et al (2010) Conserved antagonism between JMJD2A/KDM4A and HP1gamma during cell cycle progression. Mol Cell 40: 736–748. 10.1016/j.molcel.2010.11.008 21145482

[34] Armstrong JA, Papoulas O, Daubresse G, Sperling AS, John TL, Matthew PS, et al (2002) The *Drosophila* BRM complex facilitates global transcription by RNA polymerase II. EMBO J 19: 5245–5254. 10.1093/emboj/cdf517 PMC129039 12356740

[35] Qi Y, Teng Z, Gao L, Wu S, Huang J, Ye G, et al (2015) Transcriptome analysis of an endoparasitoid wasp *Cotesia chilonis* (Hymenoptera: Braconidae) reveals genes involved in successful parasitism. Arch Insect Biochem Physiol 88: 203–221. 10.1002/arch.21214 25336406

