# Supplementary material for: Characterization of joining sites of a viral histone H4 on host insect chromosomes
Source: PLoS One. 2017 May 9;12(5):e0177066. doi: 10.1371/journal.pone.0177066 (PMC5423620; doi:10.1371/journal.pone.0177066)
Supplement: S1 Table — Treatments includes in vivo transient expression of CpBV-H4 (‘vH4’) or truncated CpBV-H4 (‘vH4T’), parasitized larvae at day 7 (‘P7’) or nonparasitized larvae at day 5 (‘NP5’). (DOCX) [file pone.0177066.s001.docx]

**S1 Table**. **RNA-Seq summary of different treatment groups of *P. xylostella* larvae.** Treatments includes *in vivo* transient expression of CpBV-H4 (‘vH4’) or truncated CpBV-H4 (‘vH4T’), parasitized larvae at day 7 (‘P7’) or nonparasitized larvae at day 5 (‘NP5’).

| Treatment  groups | Trimmed  reads  (bp) | Mapped  reads (bp) | Mapping  ratios (%) |
| --- | --- | --- | --- |
| vH4 | 68,015,326 | 35,267,386 | 51.9 |
| vH4T | 60,460,538 | 31,523,829 | 52.1 |
| P7 | 108,508,176 | 28,240,047 | 26.0 |
| NP5 | 148,458,058 | 81,876,641 | 55.2 |
