# Supplementary material for: Characterization of joining sites of a viral histone H4 on host insect chromosomes
Source: PLoS One. 2017 May 9;12(5):e0177066. doi: 10.1371/journal.pone.0177066 (PMC5423620; doi:10.1371/journal.pone.0177066)
Supplement: S2 Table — The prediction used MOTIF program (www.genome.jp/tools/motifs/). (DOCX) [file pone.0177066.s002.docx]

**S2 Table. Functional prediction of repeat motifs found in CpBV-H4 joining sites.** The prediction used MOTIF program (www.genome.jp/tools/motifs/).

| Repeats | Binding proteins | Prediction parameter values |
| --- | --- | --- |
| GT-repeat | SWI-SNF_Ssr4, Fungal domain | E-value (0.30), pfam |
| AC-repeat | Gpos_C8_like, putative immunity protein/ bacteriocin | E-value (0.073), InterPro |
| CT-repeat | 2-aminoethylphosphonate ABC transporter substrate-binding protein | E-value (0.073), NCBI |
| AG-repeat | DNA polymerase III subunits  Or, Transcription elongation factor | E-value (2e-04), NCBI  E-value (0.34), NCBI |
| CAT-repeat | DNA topoisomerase III | E-value (0.002), NCBI |
| TGA-repeat  TACA-repeat  TGAG-repeat  TCAC-repeat  GTCT-repeat  TCTG-repeat  TAGA-repeat  ATTCT-repeat  TTCTG-repeat  CAATA-repeat | Collagen triple helix repeat  DNA polymerase III subunits  Ca2+-dependent lipid-binding protein  Variant erythrocyte surface antigen-1  Gly-zipper YMGG-like  Fragile site-associated protein C-terminus  transcription termination factor Rho  Predicted solute binding protein  Predicted solute binding protein  Mediator complex subunit (Med3) | E-value (0.003), pfam  E-value (4e-04), NCBI  E-value (0.002), NCBI  E-value (0.058), pfam  E-value (0.017), pfam  E-value (0.030), pfam  E-value (3e-04), NCBI  E-value (6e-04), NCBI  E-value (0.002), NCBI  E-value (0.003), pfam |
