# Supplementary material for: Characterization of joining sites of a viral histone H4 on host insect chromosomes
Source: PLoS One. 2017 May 9;12(5):e0177066. doi: 10.1371/journal.pone.0177066 (PMC5423620; doi:10.1371/journal.pone.0177066)
Supplement: S3 Table — The CpBV-H4 location was determined by the most significant ChIP assay. The locations of gene body (‘GB’), UPSTREAM (‘UP’), and downstream (‘DOWN’) were with respect to the nearest coding DNA sequence (CDS). (DOCX) [file pone.0177066.s003.docx]

**S3 Table**. **Influence of distribution of *CpBV-H4* joining sites on expression of nearest genes.** The *CpBV-H4* location was determined by the most significant ChIP assay. The locations of gene body (’GB’), UPSTREAM (‘UP’), and downstream (‘DOWN’) were with respect to the nearest coding DNA sequence (CDS).

| Scaffold ID | CDS | Major location of ChIP | Expression | Scaffold ID | CDS | Major location of ChIP | Expression |
| --- | --- | --- | --- | --- | --- | --- | --- |
| LG-01/ Sc-59 | UK2 | DOWN | DOWN | LG-14/ Sc-37 | NeR | DOWN | DOWN |
|  | SNMP | GB | DOWN |  | RBP | DOWN | DOWN |
|  | UK3 | DOWN | UP |  | UK1 | GB | UP |
|  | Mito-1 | UP | DOWN |  | WDR | UP | UP |
|  | Mito-2 | DOWN | UP |  | Whirlin | UP | DOWN |
| LG-05/ Sc-170 | Cad | DOWN | UP | LG-17/ Sc-33 | UK1 | DOWN | UP |
|  | UK2 | DOWN | DOWN |  | ThyK | UP | DOWN |
|  | CP | GB | DOWN |  | PP | GB | UP |
|  | EcoA | DOWN | UP |  | PK | DOWN | Down |
|  | UK3 | DOWN | UP |  | UK1 | UP | UP |
| LG-05/ Sc-294 | MicroSP | GB | UP | LG-21/ Sc-140 | LysoP | UP | DOWN |
|  | UK1 | UP | DOWN |  | Con-2 | UP | UP |
|  | CP | UP | UP |  | H1G1 | DOWN | UP |
|  | UK2 | UP | UP |  | X. dehy | UP | DOWN |
|  | UK3 | DOWN | UP |  | ExoCx | DOWN | DOWN |
| LG-05/ Sc-294 | UK2 | DOWN | UP | LG-22/ Sc-16 | UK2 | UP | UP |
|  | UK3 | GB | DOWN |  | UK3 | DOWN | UP |
|  | UK4 | DOWN | DOWN |  | UK4 | GB | UP |
|  | Chemo-SR | UP | UP |  | UK5 | DOWN | DOWN |
|  | Rab-II | DOWN | UP |  | UK6 | UP | DOWN |
| LG-06/ Sc-159 | Sh2D | UP | UP | LG-22/ Sc-147 | Ankyn | UP | UP |
|  | G123380 | DOWN | UP |  | S.carr | UP | UP |
|  | UK1 | UP | DOWN |  | PolyC | GB | DOWN |
|  | TEL2 | GB | UP |  | PolyC | UP | UP |
|  | FAM185A | DOWN | DOWN |  | TPser | UP | DOWN |
| LG-07/ Sc-437 | GluR | DOWN | DOWN | LG-24/ Sc-236 | UK1 | DOWN | UP |
|  | Actin | UP | DOWN |  | B4NYSO | UP | UP |
|  | ZnF | DOWN | UP |  | ZnF | GB | DOWN |
|  | UK1 | DOWN | DOWN |  | S6Hy | DOWN | DOWN |
|  | SerP | UP | UP |  | N.receptor | UP | DOWN |
| LG-12/ Sc-287 | Myosin | UP | UP | LG-26/ Sc-388 | DNA-RF | UP | DOWN |
|  | PrTu | UP | UP |  | SnRNA | DOWN | UP |
|  | GJ16239 | GB | UP |  | Zn-F | GB | UP |
|  | GlyH | DOWN | UP |  | Sfn | UP | DOWN |
|  | Tyro | DOWN | DOWN |  | IsoD | UP | DOWN |
| LG-12/ Sc-287 | VATPs | UP | DOWN |  |  |  |  |
|  | Glu-R | UP | UP |  |  |  |  |
|  | MDC | GB | DOWN |  |  |  |  |
|  | GO-R | DOWN | UP |  |  |  |  |
|  | GE19652 | DOWN | UP |  |  |  |  |
