# Supplementary material for: Characterization of joining sites of a viral histone H4 on host insect chromosomes
Source: PLoS One. 2017 May 9;12(5):e0177066. doi: 10.1371/journal.pone.0177066 (PMC5423620; doi:10.1371/journal.pone.0177066)
Supplement: S4 Table — (DOCX) [file pone.0177066.s004.docx]

**S4 Table**. **Full name and their acronyms used in Fig 6**

| Acronym | Full Name | Acronym | Full Name |
| --- | --- | --- | --- |
| AcE | Acetyl cholinesterase | PHD | PHD and RING finger domain |
| Actin | Actin-related protein 10 | PolyC | Polycomb protein eed-A |
| Ankyn | Ankyrin repeat and KH domain-containing protein | EcoA | Probable trans-2-enoyl-CoA reductase |
| Ankyn | Ankyrin repeat domain-containing protein 17 | Fam | Protein FAM185A |
| CenP | CCR4-NOT transcription complex subunit 2 | PP | Protein penguin |
| CleP | Cleft lip and palate transmembrane protein | PrTu | Protein turtle |
| Cond2 | Condensin-2 complex subunit H2 | Ch-R | Putative chemosensory receptor 21 |
| DNAf | DNA fragmentation factor subunit beta | Dehy | dehydrogenase E1 component subunit β |
| DNAr | DNA replication licensing factor Mcm6 | RabII | Rab11 family-interacting protein 1 |
| PK | Dual specificity mitogen-activated protein kinase | RalG | Ral guanine nucleotide dissociation stimulator |
| EhD | EH domain-containing protein 1 | RBP | Reticulocyte-binding protein 2 homolog a |
| F.Tre | Facilitated trehalose transporter Tret1 | R.virus | Retrovirus-related Pol polyprotein |
| Gal | Galactosylgalactosylxylosylprotein 3-beta-glucuronosyltransferase S | SolC | Solute carrier family 35 member F4 |
| GD17742 | GE19652 (Protein kinase like domain) | SMNP | Sensory neuron membrane protein 1 |
| GI23380 | GI23380 (Mitochondria eating protein) | Ser/P | Serine protease persephone |
| GJ16235 | GJ16235 (Immunoglobulin like domain) | Ser/Tre | Serine/threonine-protein kinase |
| GluR | Glutamate [NMDA] receptor subunit 1 | Sh2D | Sh2 domain containing protein, putative |
| Cat | Cadherin | S.acid | Sialic acid synthase |
| GlyP | Glycoprotein hormone beta-5 | SidF | Sideroflexin-1 |
| GO-R | Gustatory and odorant receptor 24 | sGluT | Small glutamine-rich tetratricopeptide |
| H1G1 | HIG1 domain family member 2A | snRNA | snRNA-activating protein complex subunit |
| IsoD | Isocitrate dehydrogenase [NAD] subunit beta | Suc6P | Sucrose-6-phosphate hydrolase |
| LysoP | Lysosomal aspartic protease | TEL-2 | TEL2-interacting protein 1 homolog |
| MsP | Microspherule protein 1 | ThyK | Thymidylate kinase |
| CpdP | Minor capsid protein C | TF | Tyrosine phosphatase serine |
| MitoP | Mitochondrial glutamate carrier 1 | Tyro | Tyrosine |
| Mito1 | Mitoferrin-1 | UK | Unknown function |
| Mito2 | Mitoferrin-2 | vATPs | V-type proton ATPase subunit E |
| NeRp | MMS19 nucleotide excision repair protein homolog | WDrep | WD repeat-containing protein 59 |
| Cad | Mutant cadherin | whirlin | Whirlin |
| Myon | Myoneurin | X.dehy | Xanthine dehydrogenase |
| Myosin | Myosin-2 essential light chain | ZnF | Zinc finger protein 300 |
| N.R | Neuropeptide FF receptor 1 | 39sRP | 39S ribosomal protein L42, mitochondrial |
