# Supplementary figures and images for: Characterization of joining sites of a viral histone H4 on host insect chromosomes
Source: PLoS One. 2017 May 9;12(5):e0177066. doi: 10.1371/journal.pone.0177066 (PMC5423620; doi:10.1371/journal.pone.0177066)

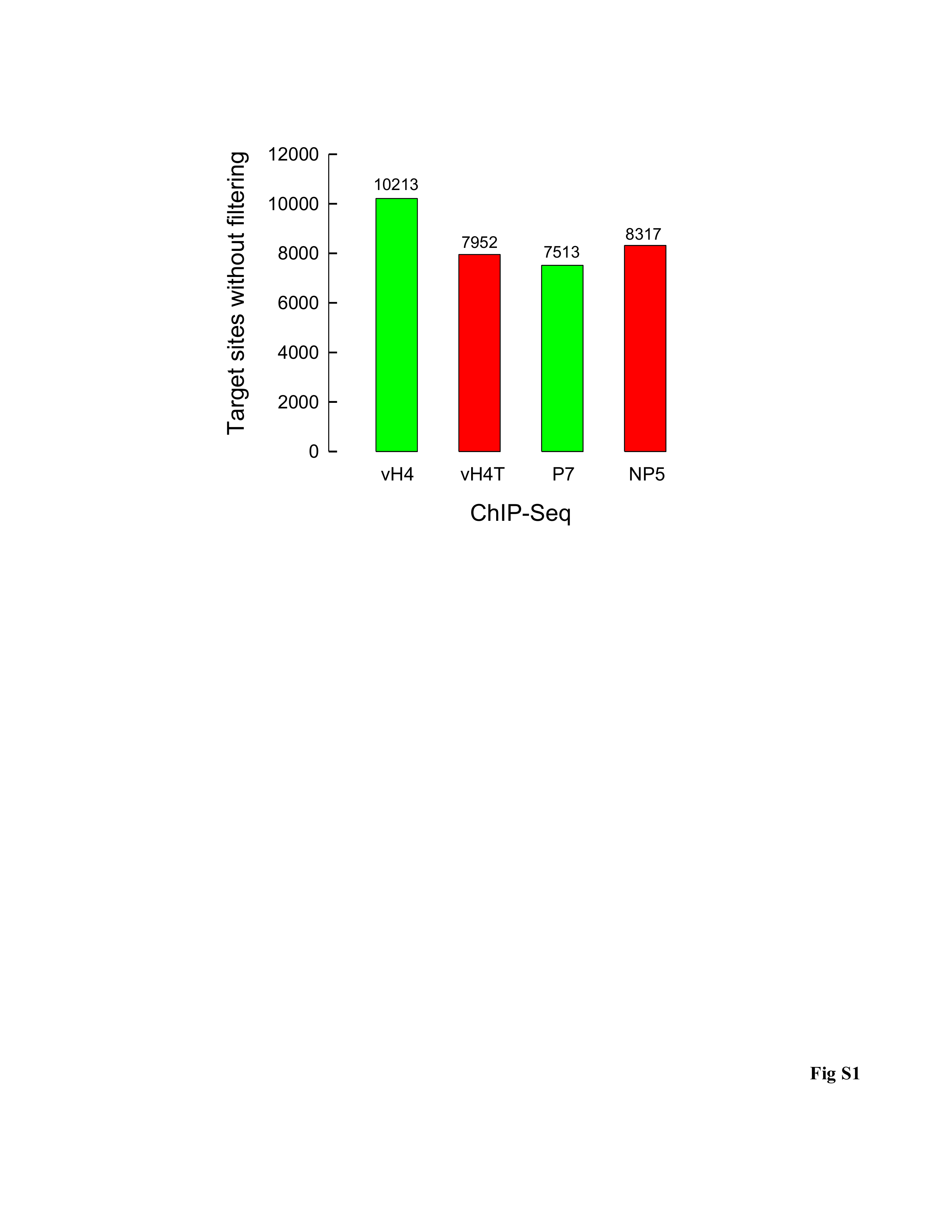

Supplement: S1 Fig — (TIF) [file pone.0177066.s005.tif]

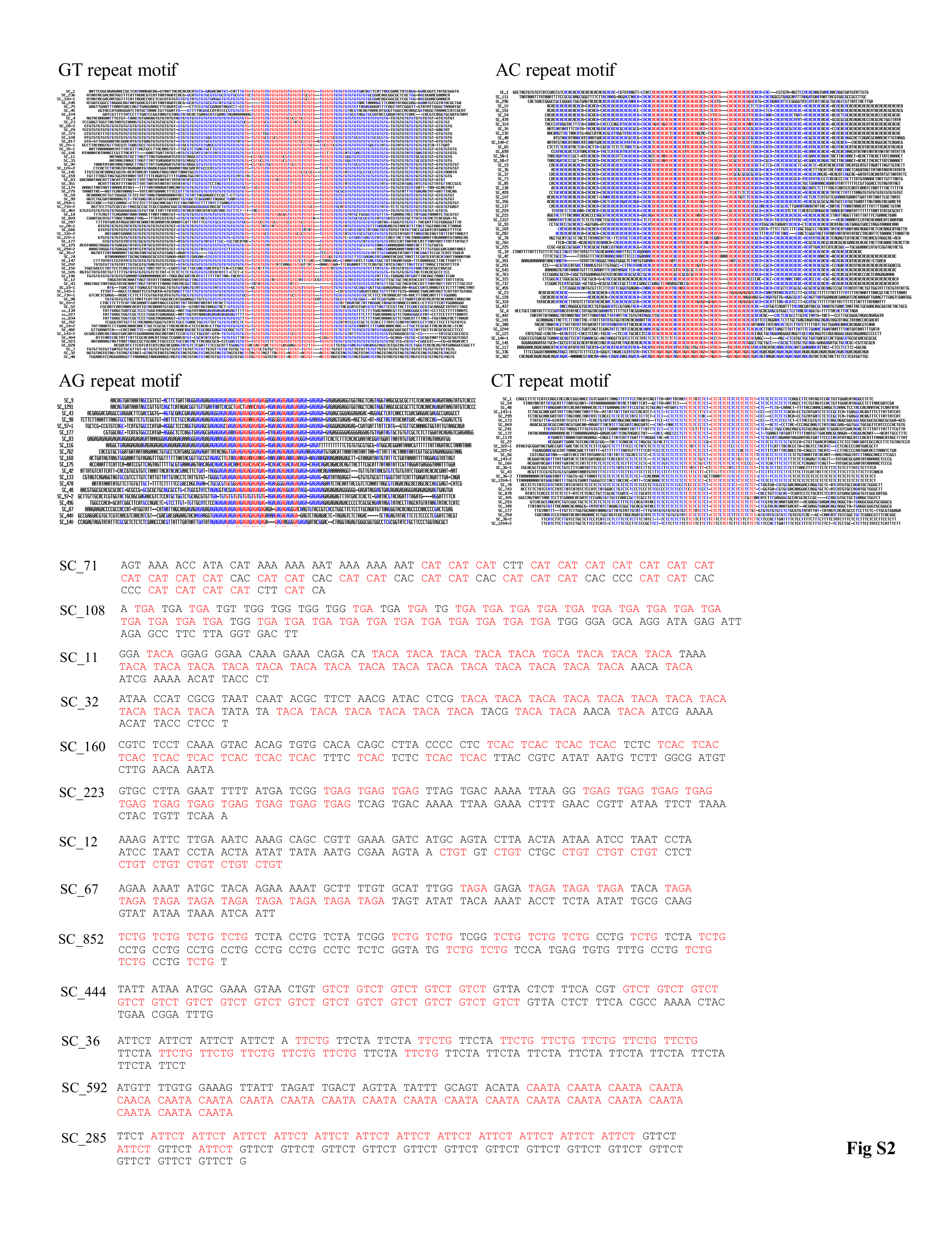

Supplement: S2 Fig — (TIF) [file pone.0177066.s006.tif]
